# Recreating Stable *Brachypodium hybridum* Allotetraploids by Uniting the Divergent Genomes of *B*. *distachyon* and *B*. *stacei*

**DOI:** 10.1371/journal.pone.0167171

**Published:** 2016-12-09

**Authors:** Vinh Ha Dinh Thi, Olivier Coriton, Isabelle Le Clainche, Dominique Arnaud, Sean P. Gordon, Gabriella Linc, Pilar Catalan, Robert Hasterok, John P. Vogel, Joseph Jahier, Boulos Chalhoub

**Affiliations:** 1 Organization and evolution of complex genomes, Institut National de la Recherche agronomique, Université d’Evry Val d’Essonne, Evry, France; 2 Unité Mixte de Recherches INRA, Agrocampus Rennes—Université Rennes 1, Institut de Génétique, Environnement et Protection des Plantes, Le Rheu, France; 3 DOE Joint Genome Institute, Walnut Creek, United States of America; 4 Agricultural Institute, Centre for Agricultural Research, Hungarian Academy of Sciences,Martonvásár, Brunszvik u 2, Hungary; 5 Department of Agriculture and Environmental Sciences, High Polytechnic School of Huesca, University of Zaragoza, Huesca, Spain; 6 Department of Botany, Institute of Biology, Tomsk State University, Tomsk, Russia; 7 Department of Plant Anatomy and Cytology, Faculty of Biology and Environmental Protection, University of Silesia,Katowice, Poland; 8 Department of Plant and Microbial Biology, University of California, Berkeley, United States of America; 9 Institute of System and Synthetic Biology, Genopole, Centre National de la Recherche Scientifique, Université d’Evry Val d’Essonne, Université Paris-Saclay, Evry, France; Ben-Gurion University, ISRAEL

## Abstract

*Brachypodium hybridum* (2n = 30) is a natural allopolyploid with highly divergent sub-genomes derived from two extant diploid species, *B*. *distachyon* (2n = 10) and *B*. *stacei* (2n = 20) that differ in chromosome evolution and number. We created synthetic *B*. *hybridum* allotetraploids by hybridizing various lines of *B*. *distachyon* and *B*. *stacei*. The initial amphihaploid F1 interspecific hybrids were obtained at low frequencies when *B*. *distachyon* was used as the maternal parent (0.15% or 0.245% depending on the line used) and were sterile. No hybrids were obtained from reciprocal crosses or when autotetraploids of the parental species were crossed. Colchicine treatment was used to double the genome of the F1 amphihaploid lines leading to allotetraploids. The genome-doubled F1 plants produced a few S1 (first selfed generation) seeds after self-pollination. S1 plants from one parental combination (Bd3-1×Bsta5) were fertile and gave rise to further generations whereas those of another parental combination (Bd21×ABR114) were sterile, illustrating the importance of the parental lineages crossed. The synthetic allotetraploids were stable and resembled the natural *B*. *hybridum* at the phenotypic, cytogenetic and genomic levels. The successful creation of synthetic *B*. *hybridum* offers the possibility to study changes in genome structure and regulation at the earliest stages of allopolyploid formation in comparison with the parental species and natural *B*. *hybridum*.

## Introduction

Polyploidy, or to whole genome duplication, is a major evolutionary force in eukaryotes and is particularly prominent and recurrent in angiosperms [1–8]. Allopolyploids combine two or more divergent homoeologous genomes, usually through interspecific or intergeneric hybridization, accompagnied by chromosome doubling. In contrast, autopolyploids combine genomes from the same species or even the same individual. Over the past few decades, numerous studies have shown that genetic, epigenetic and/or gene expression changes are common consequences of polyploidization across a wide range of species [7, 9–15]. The extent, importance, ‘timing’ and mechanisms by which these changes occur depend on the allopolyploid model,the parental lineages crossed and the genome-doubling process (e. g., from crosses between stable autotetraploids or from the doubling of amphihaploid interspecific hybrids) [8, 16–18].

The Poaceae is one of the largest angiosperm families comprising more than 12,000 species, classified into 771 genera [19, 20]. In this family, the tribe Brachypodieae diverged from Aveneae and Triticeae about 38 million years ago (Mya), whilst tribe Ehrhartoideae (rice) diverged approximately 55 (49–66) Mya [21, 22]. The intermediate phylogenetic position of Brachypodieae between tropical cereals like *Sorghum* and *Zea* (Panicoideae) and *Oryza* (Ehrhartoideae) and temperate grasses such as wheat (*Triticum* and *Aegilops*) and barley (*Hordeum*) [22–25] led to establishing *Brachypodium distachyon* as a model organism for temperate grasses [26–28].

Investigations of about 20 known *Brachypodium* taxa revealed seven diploid species with variable basic chromosome numbers (x = 5, 8, 9, 10) that have hybridized with each other or with ancestral species to form at least seven allopolyploid species whereas the ploidy level of six other extant species has not been investigated yet [24, 29, 30]. This cytological diversity and large dysploidy makes *Brachypodium* an ideal model to study the evolution of chromosomes and their basic number within a genus [20] as well as the consequences of allopolyploidy.

**Fig 1:**
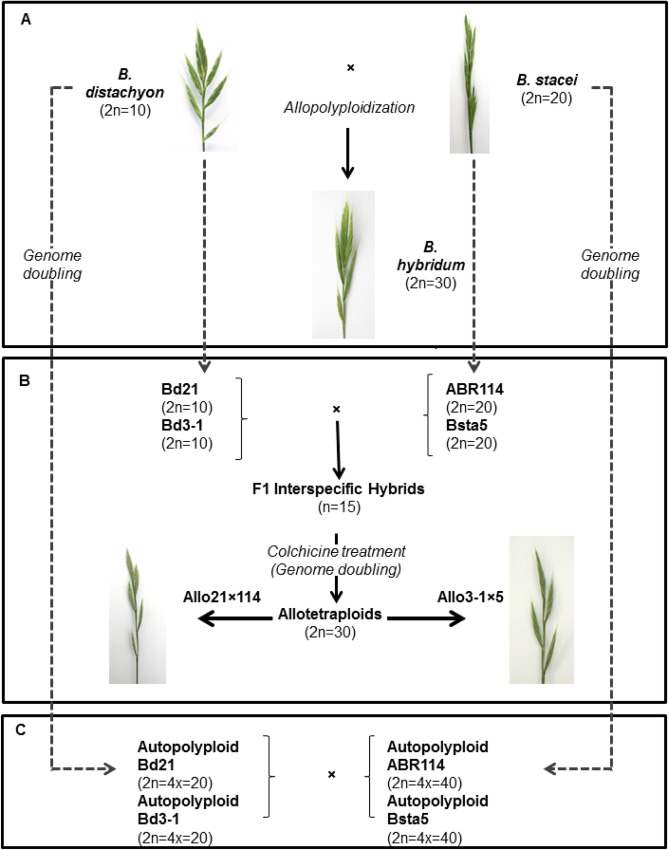
The *Brachypodium* polyploid model. (A) Evolution and origin of *B*. *hybridum* allotetraploid through natural hybridizations between the diploid species *B*. *distachyon* and *B*. *stacei*. (B-C) Strategies for synthesis of F1 interspecific hybrids and allotetraploids either by direct crossing of diploid accessions (B) (lines used are indicated) or by doubling their genomes to obtain the respective autotetraploids, followed by interspecific crossing (C).

The annual species *B*. *distachyon* (2n = 10) has the lowest chromosome number and is thought to have played a pivotal role in the evolution of the genus through interspecific hybridization and the formation of polyploid species [24, 30, 31]. Comparative cytogenetic and molecular analyses showed that *B*. *distachyon*’s large chromosomes likely originated via descending dysploidy, acting as fusions of smaller chromosomes of a putative ancestral *Brachypodium* species, very close to *B*. *stacei* (2n = 20) [31–33]. Within this framework, the allotetraploid *B*. *hybridum* (2n = 30) was derived through interspecific hybridization between *B*. *distachyon* and *B*. *stacei* approximately one Mya [33] (Fig 1). Together, these three species comprise an excellent model to investigate the impact of polyploidization on the organization and evolution of plant genomes, because they possess small genomes, obvious morphological differences and the genomes of *B*. *distachyon* and *B*. *stacei* (and by corollary the sub-genomes of *B*. *hybridum*) are quite divergent in terms of sequence and chromosome number [31–34]. Further supporting this system, the genomes of all three species have been sequenced: the *B*. *distachyon* genome sequence was first published in 2010 [28] and has recently been improved to an essentially ‘finished’ genome sequence with the only remaining ambiguity being the location of some centromeric repeats (https://phytozome.jgi.doe.gov/pz/portal.html#!info?alias=Org_Bdistachyon_er), the first draft of the *B*. *stacei* is available (https://phytozome.jgi.doe.gov/pz/portal.html#!info?alias=Org_Bstacei) and a high quality assemble has been created for *B*. *hybridum* (Vogel unpublished). In addition all three species are small, self-fertile and experimentally tractable [35].

Several pathways for synthesizing allotetraploids have been proposed [36, 37]. The one-step model suggests that allopolyploids can be formed by a direct hybridization, either by the fusion of unreduced gametes, which are produced at low frequencies in diploid species, or the hybridization between two different autotetraploid species. By contrast, the two-step model requires the formation of amphihaploid interspecific hybrids from reduced gametes of two different species followed by chromosome doubling [38, 39]. Synthesizing allotetraploids by applying the two-step model has been successfully reported for various species from different genera [15, 40–43]. Allotetraploids have also been synthesized by a variant of the one step method where the genomes of the parental species were artificially doubled by colchicine treatment to form autotetraploids. These autotetraploids, each producing 2n gametes, were then hybridized to obtain allotetraploids [40, 44–46].

The aim of this study is to further develop a *Brachypodium* polyploid model system by synthesizing *Brachypodium* allotetraploids, through hybridization between *B*. *distachyon* and *B*. *stacei* (Fig 1), and characterizing their stability at the genomic, phenotypic and cytogenetic levels in comparison to the parental species and the natural *B*. *hybridum*.

## Materials and Methods

### Plant material and growth

Six inbred lines of three *Brachypodium* species: *B*. *distachyon* (Bd21 and Bd3-1), *B*. *stacei* (ABR114 and Bsta5) and *B*. *hybridum* (ABR113 and Bhyb30) were used in this study (Fig 1A and 1B) [33, 47]. Autotetraploid plants of *B*. *distachyon* lines Bd21 and Bd3-1 and *B*. *stacei* lines ABR114 and Bsta5 were generated in our lab (Vinh Ha Dinh Thi and Boulos Chalhoub, unpublished) (Fig 1C).

After removal of the lemmas and paleas, seeds were surface sterilized using a 10% bleach solution containing a drop of Tween-20 for three minutes. The seeds were then rinsed in sterile water three times. Germination was synchronized by incubating the seeds in Petri dishes at 4°C for 3 days, and then at room temperature for five days. The seedlings were transferred into pots (10×7 cm) containing a mixture of equal volumes of peat moss and sand supplemented with a slow release fertilizer (2 g/L, Osmocote® Standard 14-14-14, Scotts-Sierra Horticulture, Marysville, OH, USA). Greenhouse conditions were set at day temperature of 22°C and night temperature of 18°C, with a 16 h photoperiod.

### Vegetative propagation

F1 interspecific hybrids, the colchicine-treated F1 (S0), and the S1 allotetraploids were vegetatively propagated to create large numbers of plants from these sterile or nearly sterile lines (S1 Fig). Root development from secondary tillers was stimulated by covering the base of the plants with soil and adding solution 0.25% of the auxin indole-3-butyric Acid (IBA) [48] to the irrigation solution. Two to three weeks later, secondary tillers with roots were removed, cut and placed in new pots (S1C and S1D Fig).

### Interspecific crossing between *B*. *distachyon* and *B*. *stacei*

We tried to generate synthetic allotetraploids by interspecific hybridization between *B*. *distachyon* and *B*. *stacei*. Two different lines of each species, together with their derived autotetraploids, that we created previously (Vinh Ha Dinh Thi and Boulos Chalhoub, unpublished), were used (Fig 1). While hybridizing diploid lines from the two species would create amphihaploid (n) F1 hybrids (Fig 1B), crossing the autotetraploid lines would lead directly to (2n) hybrids, i.e. alloteraploids (Fig 1C).

Reciprocal crosses between diploid *B*. *distachyon* (lines Bd21 and Bd3-1) and *B*. *stacei* (lines ABR114 and Bsta5) were performed over four years (2011–2014) in the spring and fall seasons, the best seasons for flowering and pollination in our greenhouses. Flowering time was variable among lines to be crossed. Thus, in order to ensure simultaneous flowering, multiple sowings were done for each of the lines at 15 day intervals (from January to March).

**Fig 2:**
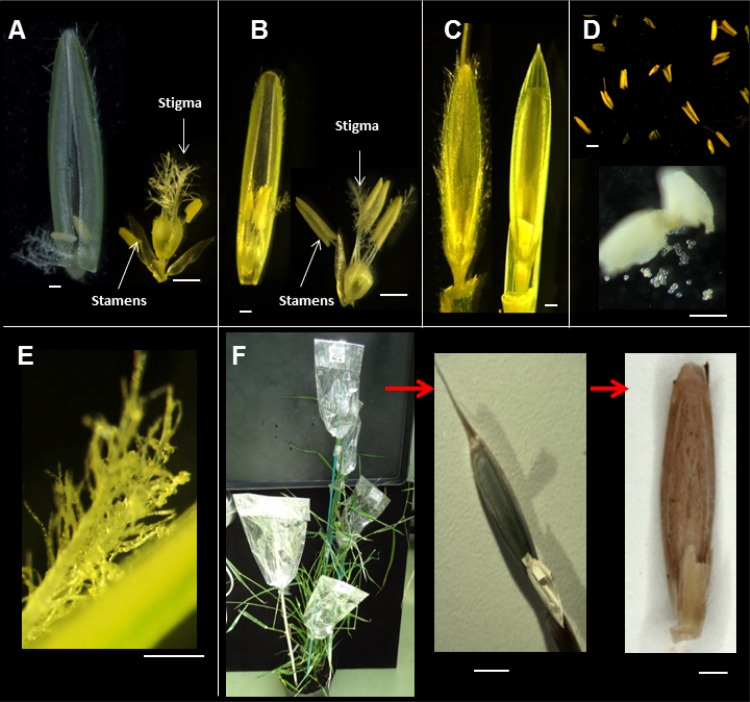
Interspecific hybridization in *Brachypodium*. (A) Floret structure of diploid *B*. *distachyon* line Bd3-1 at the stage used for crossing. Note the presence of two stamens. (B) Floret structure of diploid *B*. *stacei* line Bsta5 at the optimal stage for crossing. Note the presence of three stamens. (C) Emasculated florets of Bd3-1 (left) and Bsta5 (right). (D) Ripe anthers (Bsta5 shown) were placed on a microscope slide (above), and some dehisced 15–20 minutes later (below). (E) Stigma (Bd3-1 shown) with the pollen grains after pollination. (F) All other florets were removed from the inflorescences that were bagged to avoid unwanted cross-pollination. Production of putative hybrid seed was observed after 15 days and mature hybrid seeds were collected when completely dry (shown here without palea and lemma). Bars represent = 1mm.

Emasculation and pollination methods were adapted from Steinwand and Vogel (http://jgi.doe.gov/our-science/science-programs/plant-genomics/brachypodium/). Emasculations were accomplished by removal of the indehiscent anthers from the female parent plants on the two or three basal florets of the spikelet in the morning (10:00 am to midday, Fig 2A–2C). The emasculated flowers were bagged (NatureflexTM 70x130mm bags) to avoid contamination by non-selected pollen. Pollen from the selected paternal parent was collected from the male parent in the afternoon of the same day or one day later by placing nearly ripe anthers on a glass slide for 5–10 min. Most ripe anthers became more turgid and some of them dehisced on the slide (Fig 2D). Pollen grains were transferred to the emasculated flowers (Fig 2E) and the pollinated inflorescences were bagged (Fig 2F) to avoid pollination by stray pollen. Seed formation was recorded 5 to 6 days after pollination

The putative F1 amphihaploid interspecific hybrid seeds were collected at maturity (at least 4 weeks after pollination). The seeds were kept at 4°C for three weeks and then at room temperature for two months. They were germinated to produce plants as described above. True interspecific hybrid plants were validated through cytological analysis and PCR markers.

All F1 amphihaploid interspecific hybrid plants were vegetatively propagated as described above (S1 Fig and S1 Table). The propagated plants were split into two groups. One group was grown in a greenhouse without colchicine treatment as control to see if spontaneous chromosome doubling would restore fertility and lead to the production of allotetraploid seeds. The second group was treated with colchicine to induce chromosome doubling.

### Colchicine treatment of F1 interspecific hybrids

We treated plants with colchicine using a protocol adapted from the method described by Jahier [49], which was successfully used for wheat [43].

Vegetatively-propagated plants at 4–5 leaf stage were completely immersed for three hours in an aqueous solution of colchicine (Sigma-Aldrich Co., cat. no. C9754), at concentrations of 2.5 g/l, 5g/l or 7.5 g/l, and containing 2% DMSO (dimethyl sulfoxide, Sigma-Aldrich Co., cat. no. D8418). The colchicine-treated plants were then transplanted into fresh soil, without rinsing, and grown in a greenhouse. By seven to ten days after treatment surviving plants were producing new growth. Necrotic lesions observed on the treated leaves suggested that the treatment was effective.

### Flow cytometry (FCM) analysis

FCM was used to determine the ploidy level in F1 hybrids and synthetic allopolyploids. Calibration was done using the profiles and the C-values previously determined for *B*. *stacei* (0.564 pg/2C), *B*. *distachyon* (0.631pg/2C) and *B*. *hybridum* (1.265 pg/2C) [33]. Analyses were performed on young leaves obtained from plants grown in the greenhouse as described in [50–52].

### Chromosome counting and chromosome Fluorescent *in situ* hybridization (FISH)

Preparation of slides and hybridization using bacterial artificial chromosome—fluorescent *in situ* hybridization (BAC-FISH) was carried as described in [53–55]. A *B*. *distachyon* BAC clone, ABR1-63-E6, that hybridizes all chromosomes of *B*. *distachyon*, but not those of *B*. *stacei* was labelled by random priming with biotin-14-dUTP (Invitrogen, Life Technologies) [56]. A ribosomal DNA probe, pTa 71,[57] which contains a 9-kb *Eco*RI fragment of rDNA repeat unit (18S-5.8S-26S genes and spacers) isolated from *Triticum aestivum* was labelled with Alexa-488 dUTP by random priming. Biotinylated probe was immunodetected using Texas Red avidin DCS (Vector Laboratories) and the signal was amplified with biotinylated anti-avidin D (Vector Laboratories). The chromosomes were mounted and counterstained in Vectashield (Vector Laboratories) containing 2.5 μg/mL 4’,6-diamidino-2-phenylindole (DAPI). Fluorescence images were captured using a CoolSnap HQ camera (Photometrics, Tucson, Ariz) on an Axioplan 2 microscope (Zeiss, Oberkochen, Germany) and analysed using MetaVue^TM^ (Universal Imaging Corporation,Downington, PA).

### Estimation of pollen abundance and viability

To estimate pollen viability, anthers were sampled the day of anthesis and pollen was stained with acetocarmine as described in [49]. Mature anthers were macerated in a drop of acetocarmine to release the pollen grains and pollen viability was estimated based on the amount of stain taken up under light microscope. Viable pollen grains appear dark purple because they take up acetocarmine whereas non-viable pollen grains do not take up acetocarmine and appear light.

### DNA marker development and analysis

Genomic DNA was extracted from young leaves sampled as described previously [58].

Two types of polymorphic markers were used, simple sequence repeats (SSRs) and gene sequence-derived markers.

Twenty-two SSR markers were chosen from previous studies (S2 Table) as descrived in [59–62]. Four of these (ALB165, ALB311, BdSSR330 and R2-3-ABI) were previously shown to discriminate *B*. *distachyon*, *B*. *stacei* and *B*. *hybridum* [59].

Markers were also developed from orthologous *B*. *distachyon* and *B*. *stacei* gene sequences (early release access of the *B*.*stacei* genome is available through Phytozome (https://phytozome.jgi.doe.gov/pz/portal.html)Pairs of orthologous genes with 6–30 bp insertion/deletion polymorphisms, based on sequence alignments, were selected. Conserved PCR primers flanking the indels were designed using Primer3 (http://biotools.umassmed.edu/bioapps/primer3_www.cgi). A total of 149 primer pairs were designed for 134 orthologous genes distributed along all five *B*. *distachyon* chromosomes (S2 Table).

All SSR and gene-derived markers were tested to determine if they were polymorphic between the *B*. *distachyon* and *B*. *stacei* lines used in this study. In addition, they were also tested to make sure they amplify both the *B*. *distachyon* and the *B*. *stacei* sized bands on DNA from *B*. *hybridum* (lines ABR113 and Bhyb30) or a mixture of equal amount of *B*. *distachyon* and *B*. *stacei* DNA (Bd21 and ABR114; Bd3-1 and Bsta5). For the fertile allotetraploid allo3-1×5, the S1 plant and 118 plants from S2 generation were analyzed.

A marker was considered rearranged in a synthetic allopolyploid plant if its PCR amplification pattern was different from that observed in the mixture of parental DNA and/or sister allopolyploid plants from the same generation [43].

Polymerase chain reactions (PCR) were performed according to [58], in a 10 μl final volume with 200 μM of each deoxynucleoside triphosphate (dNTP), 500 nM of each primer, 0.2U of Taq DNA polymerase (Perkin Elmer, Norwalk, CT, USA) and 25 ng of template DNA. PCR products were separated in 3% SeaKem LE agarose gels (Lonza).

### Phenotypic analysis

Fifteen morphological characters were measured and compared between synthetic allotetraploids, *B*. *distachyon*, *B*. *stacei* and natural *B*. *hybridum* (S2 Fig and S3 Table). Three inflorescence traits that could impact seed production were recorded: number of spikelets per inflorescence, number of florets per spikelet and number of florets per inflorescence. For synthetic polyploids with low fertility, we also recorded percent of fertile florets, seed number per inflorescence and 1,000 seed weight. Five additional inflorescence characters were also measured: inflorescence length (total length and length without awns), spikelet length (total length of spikelet excluding awns and averaging all spikelet lengths per each inflorescence), the distance between two spikelets on the inflorescence (the average of all distances in one inflorescence), upper glume length and upper glume width. Four floral characters were measured, floret length, lemma length, lemma width from the basal floret, and awn length (the longest within the spikelet) (S2 Fig). At least five plants per genotype were analyzed as replicates. Statistical analysis was done using non-parametric Kruskal-wallis test [63].

## Results

We used two approaches to synthesize allotetraploids from *B*. *distachyon* and *B*. *stacei*. The first approach was to cross diploid *B*. *distachyon* and *B*. *stacei* to produce an amphihaploid F1, followed by colchicine treatment to double the chromosomes. The second approach was to first produce *B*. *distachyon* and *B*. *stacei* autotetraploid plants and then cross them. Since autotetraploids should have 2n gametes, the expected F1 progeny would be allotetraploid without need of further chromosome doubling.

### Crossing *B*. *distachyon* and *B*. *stacei* diploids

Reciprocal crosses were performed between *B*. *distachyon* and *B*. *stacei* (Fig 2). Two different diploid lines of *B*. *distachyon* (Bd21, Bd3-1) and two lines of *B*. *stacei* (ABR114 and Bsta5) were crossed (four genotype combinations) (Table 1).

A total of 9,388 crosses between the two diploid species were performed over a four year period and 122 mature seeds were obtained (Table 1). Among these, 68 were obtained from 4,587 crosses where *B*. *distachyon* was the maternal parent and 54 from 4,801 crosses where *B*. *stacei* was the female parent (Table 1). Only 38 of the 122 mature seeds (31%) germinated and produced viable plants. In comparison, the germination rates of *B*. *distachyon*, *B*. *stacei* and natural *B*. *hybridum* were usually around 96%.

**Table 1:** Interspecific crosses made between diploid *B*. *distachyon* and *B*. *stacei*.

♂♀	*B*. *stacei* ABR114	*B*. *stacei* Bsta5	*B*. *distachyon* Bd21	*B*. *distachyon* Bd3-1
***B*. *stacei* ABR114**	_*	_	2798^a^	627
			25^b^	10
			10^c^	0
			0^d^	0
***B*. *stacei* Bsta5**	_	_	530	846
			6	13
			2	3
			0	0
***B*. *distachyon* Bd21**	2664	565	_	_
	39	4		
	16	0		
	4	0		
***B*. *distachyon* Bd3-1**	541	817	_	_
	4	17		
	0	6		
	0	2		

* No crosses made between lines of the same species

^a^ number of made crosses

^b^ number of seeds obtained

^c^ number of germinated seeds

^d^ number of true F1 interspecific hybrids based on molecular markers and karyotype

To determine which of the 38 putative F1 plants were true hybrids, we first used codominant SSR markers that differentiate *B*. *distachyon* and *B*. *stacei* (see below). This analysis identified six bona-fide F1 interspecific hybrids, four arising from the 2,664 crosses between *B*. *distachyon* Bd21 and *B*. *stacei* ABR114 (designated hereafter as F1_21×114) and two from the 817 crosses between *B*. *distachyon* Bd3-1 and *B*. *stacei* Bsta5 (designated hereafter as F1_3–1×5). The final success rate for these crosses was 0.15% and 0.245%, respectively. Interestingly, we failed to obtain any true F1 interspecific hybrids from the 4,801 crosses where *B*. *stacei* was the female partner (all four genotype combinations) as well as from crosses between the two other genotype combinations where *B*. *distachyon* was the female partner (Table 1).

One F1_3–1×5 hybrid plant died before flowering. The five remaining F1 hybrids were vegetatively-propagated (S1 Fig) and separated into two batches (S1 Table). The first batch of 99 plants were grown without colchicine treatment to test if spontaneous chromosome doubling would occur and lead to fertile sectors as has been observed in other systems [43, 64]. The second batch of 226 plants was treated with colchicine to induce chromosome doubling and fertility.

**Fig 3:**
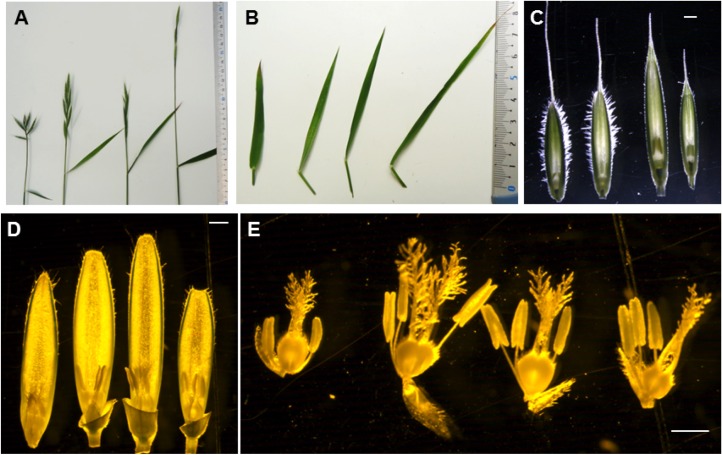
Phenotypic comparison of F1 amphihaploid hybridsnatural *B*. *hybridum*, *B*. *distachyon* and *B*. *stacei*. From left to right: *B*. *distachyon* (Bd21), the F1 amphihaploid hybrid F1_21×114, natural *B*. *hybridum* (ABR113) and *B*. *stacei* (ABR114). (A) Inflorescence morphology; (B) flag leaf; (C) lemma and palea; (D) palea, anthers and pistile, and (E) dissected florets showing anthers and stigmas. Bars: 1mm.

Overall, the F1_21×114 and F1_3–1×5 amphihaploid F1 plants were phenotypically similar to natural *B*. *hybridum* and intermediate between *B*. *distachyon* and *B*. *stacei* (e.g. inflorescence architecture and flag leaf morphology) (Fig 3A and 3B). However, for some phenotypic traits the F1s more closely resembled one of the parents. Floret hairiness and floret shape of the F1 amphihaploid hybrids were more similar to *B*. *distachyon* than to *B*. *stacei*, whereas the number of stamens and stigma structure were more similar to *B*. *stacei* (Fig 3C–3E).

#### Amphihaploid F1 interspecific hybrids were sterile

F1 interspecific amphihaploid hybrids are normally sterile, presumably because of defective chromosome pairing at meiosis [65–67]. However, in several cases amphihaploid interspecific hybrids have been reported to produce seeds, most likely by spontaneous genome doubling prior to flowering [43, 64]. We tested this possibility with 99 vegetatively propagated plants from the five different F1 interspecific hybrids. Over a period of 2 years no seeds were produced (S1 Table). Each individual plant produced about 20–30 tillers, with two to three inflorescences per tiller and an average of 33 florets per inflorescence. Thus, about 1,320 to 2,970 florets were checked for each individual plant and a total of approximately 128,040 to 288,090 florets for all F1 plants combined.

#### Chromosome doubling of amphihaploid F1 plants and generation of allopolyploids

One-hundred-fifty-three vegetatively propagated plants from the four original F1_21×114 interspecific hybrids and one of the F1_3–1×5 interspecific hybrids were treated with colchicine to induce chromosome doubling. The majority of plants treated with 2.5 g/l and 5 g/l colchicine solution survived (74% and 87% survival, respectively). By contrast, only 23% of the plants treated with 7.5 g/l colchicine solution survived (S1 Table).

**Fig 4:**
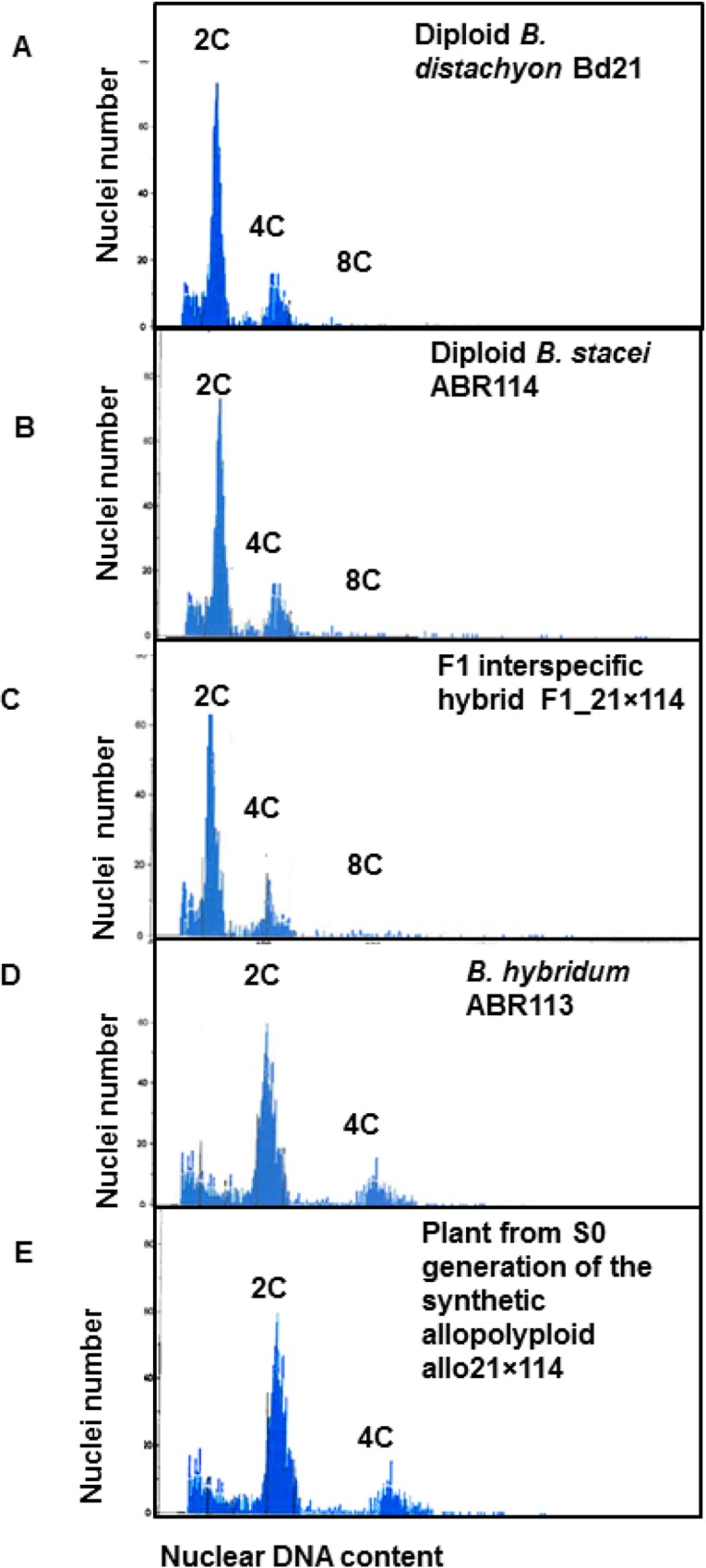
Flow cytometry (FCM) profiles. (A) Diploid *B*. *distachyon* Bd21 and (B) diploid *B*. *stacei* ABR114 showing two peaks corresponding to the G1 (2C DNA) and G2/M (4C DNA) nuclei. (C) The F1 interspecific hybrid of *B*. *distachyon* Bd21 and *B*. *stacei* ABR114, with a profile similar to that of the parental species. (D) Position of the 2C and 4C peaks for *B*. *hybridum* ABR113 indicate a nuclear genome size twice that of *B*. *distachyon* or *B*. *stacei*. (E) FCM profile of the synthetic allopolyploid allo21×114 (S0 generation) is similar to that of natural *B*. *hybridum* (D) and double the genome size of the F1 (C) and diploid parental species (A-B). The X and Y axes show relative DNA content per nuclei estimated by fluorescence intensity and the frequency-count (cell number), respectively.

We compared FCM profiles from leaves of colchicine-treated F1 interspecific hybrid plants with those of the non-treated plants and of the parental lines. The results revealed the expected average c-value of ~0.6 pg for the F1 interspecific hybrids, which is similar to the c-values of *B*. *distachyon* and *B*. *stacei*. The positions of G1 and G2 peaks in the F1 hybrids were also similar to their counterparts in the diploid parental species (Fig 4A–4C). The 24 colchicine-treated F1 interspecific hybrid plants (23 plants vegetatively-multiplied from the F1_21×114 initial plant and one from the initial F1_3–1×5 plant) showed G1 and G2 peaks at similar positions to those of the natural allotetraploid *B*. *hybridum*, indicating that the genomes of these colchicine-treated hybrids have been partially or completely doubled (Fig 4D and 4E; Table 2).

**Table 2:** Summary of cytogenetic analyses conducted on *Brachypodium distachyon* (lines Bd21 and Bd3-1), *B*. *stacei* (lines ABR114 and Bsta5), their derived autopolyploids (4× followed by same line name), F1 interspecific hybrids, synthetic allotetraploids (generations S0 to S2) and the natural *B*. *hybridum* (lines ABR113 and Bhy30).

Species/cross	Line	Flow cytometry		Mitosis metaphase stage			
		Plants tested	Ploidy level	Plants tested	Chromosome number (DAPI staining)	Number of 45S rDNA sites	Number of chromosomes hybridizing with the BAC ABR1-63E06**
B. *distachyon*	Bd21	2	2x	1	10	2	-*
	Bd3-1	2	2x	1	10	2	-
	4×Bd21	3	4x	2	20	4	-
	4×Bd3-1	2	4x	2	20	4	-
B. *stacei*	ABR114	2	2x	1	20	2	-
	Bsta5	2	2x	1	20	2	-
	4×ABR114	15	4x	15	40	4	-
	4×Bsta5	1	4x	1	40	4	-
F1 interspecific hybrids	F1_21×114	40^a^	2x	8	15	2	5
	F1_3–1×5	1^a^	2x	1	15	2	5
Synthetic allotetraploids (S0 generation)	allo21×114	23^a^	4x	10	30 (6)^b^	4(6)^b^	10 (6)^b^
					30 and 15 (4)^c^	4 and 2(4)^c^	10 and 5(4)^c^
	allo3-1×5	1^a^	4x	1	30	4	10
Synthetic allotetraploids (S1 generation)	allo21×114	10^a^	4x	5	30	4	10
	allo3-1×5	10^a^	4x	2	30	4	10
Synthetic allotetraploids (S2 generation)	allo3-1×5	10	4x	10	30	4	10
*B*. *hybridum*	ABR113	2	4x	1	30	4	10
	Bhyb30	2	4x	1	30	4	10

* Not analyzed

** Specifically hybridizes to *B*. *distachyon* chromosomes and the *B*. *distachyon*-like chromosomes in *B*. *hybridum*.

^a^ These plants were obtained by vegetative cuttings from one initial plant of each category

^b^ Number of plants showing doubled genome karyotype

^c^ Number of plants showing mixed karyotype, indicating chimerci tissues with both cellesof doubled and non-doubled genome

Metaphase chromosome counting of root-tip cells revealed that six plants derived from F1_21×114 and one plant derived from F1_3.1×5 had a chromosome number of 30 in all cells examined which is consistent with whole genome duplication. Four plants derived from F1_21×114 had variable chromosome number in different cells indicating that these plants were a mosaic of cells with doubled and non-doubled genomes (Table 2).

The seven colchicine-treated F1 plants with 30 chromosomes in all cells observed were considered to be zero-selfed (S0) generation of the allotetraploid allo21×114 derived from F1_21×114 (six plants), and of the allotetraploid allo3-1×5, derived from F1_3–1×5 (one plant). These S0 plants were maintained by vegetative propagation. Only two S1 (selfed generation subsequent to S0) seeds were obtained from more than 200,000 flowers from 153 S0 allo21×114 plants, whereas one S1 seed was obtained from the single S0 plant of allo3-1×5. This indicated an overall low fertility in the first generation of the synthetic allotetraploids. Only one of the two S1 seeds of allo21×114 and the single S1 seed of allo3-1×5 germinated and produced mature S1 plants. The S1 plants were also vegetatively propagated to produce 161 allo21×114 S1 and 48 allo3-1×5 S1 plants.

**Fig 5:**
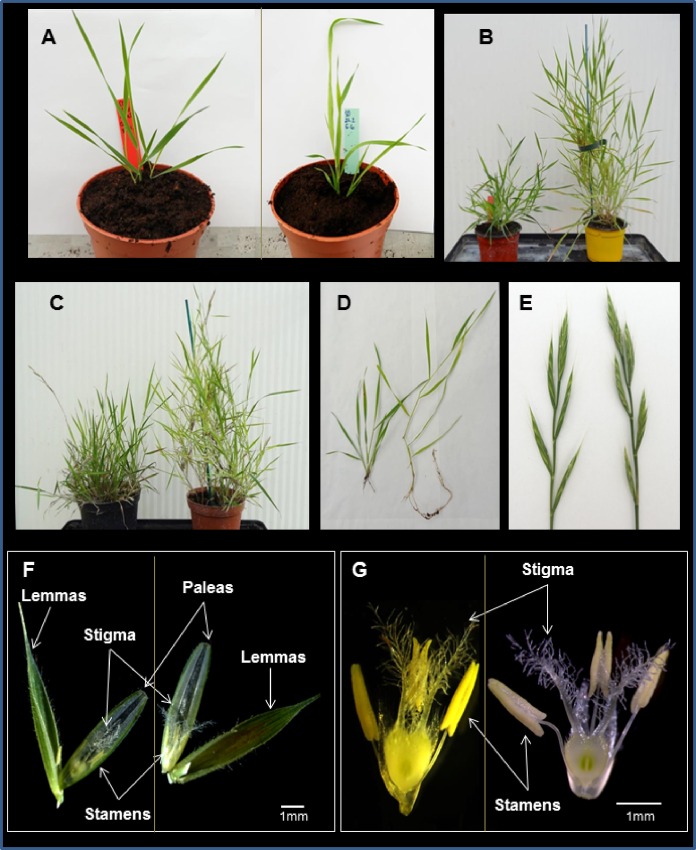
Morphology of the synthetic allotetraploids at the S1 generation: allo21×114 (right) and allo3-1×5 (left). (A) Vegetatively multiplied plants; (B) Plants at the tillering stage; (C) Flowering stage; (D) Tillers; (E) Spike structure; (F) Dissected florets, with visible lemmas, paleas, stamens and pistils; (G) floret close up. Note that differences in floret color are due to differences in lighting, not differences in actual floret color.

All S1 plants were taller and more vigorous than the F1 amphihaploid hybrids and the parental species. The two synthetic allotetraploids exhibited similar morphology during the early stages of leaf development and tillering (as defined by [68] (Fig 5A). However, they showed differences in stem elongation. Allo21×114 stems tended to have longer and more internodes, leading to taller plants, than allo3-1×5 (Fig 5B–5D). Inflorescence architecture was similar for both lines with long inflorescences and three to five spikelets (Fig 5E). Florets of both lines were also similar. They had long hairy lemmas, three stamens and feathery stigmas (Fig 5F–5G). Floral characteristics of the F1 hybrids, S1 allotetraploids, the parental lines and natural *B*. *hybridum*, are summarized in S4 Table. Genome size of the S1 allotetraploid plants was assessed by FCM and the positions of their G1 and G2 peaks (Fig 4D) were similar to those observed in natural *B*. *hybridum* (Fig 4C).

S1 plants of allo3-1×5 were fertile with 23% of florets producing seed. While this is much greater than the S0 plants, it is lower than wild *B*. *hybridum* lines ABR113 (91%) and Bhyb30 (68%) (Table S3). More than 100 S2 seeds of allo3-1×5 were sown and almost all of them germinated and produced plants for further cytogenetic and genomic characterization. Surprisingly, all 135 vegetatively propagated S1 allo21×114 plants were sterile.

#### Sterility of the synthetic allopolyploid allo21×114

In order to characterize the sterility of S1 allo21×114, we used acetocarmine staining to determine pollen shape [49]. Anthers of S1 allo21×114 plants contained few, ~15–17, normally shaped, potentially viable pollen grains, whereas anthers from natural *B*. *hybridum* typically contain ~170–200 viable pollen grains (S3 Fig). The near absence of normal pollen suggests that S1 allo21×114 plants have significantly reduced male fertility.

To examine the female fertility of S1 allo21×114 plants we pollinated emasculated S1 allo21×114 flowers (as well as F1_21×114) with pollen from the two diploid parents and two natural *B*. *hybridum* lines but no seeds were obtained (S5 Table). By comparison, 10 seeds were obtained from 35 crosses between the two natural *B*. *hy*bridum lines.

Our results suggest that S1 allo21×114 plants may be both male and female sterile.

### Crossing autotetraploid *B*. *distachyon* and *B*. *stacei*

We performed 4,384 reciprocal crosses between autotetraploids from two lines of *B*. *stacei* (ABR114 and Bsta5) and two lines of *B*. *distachyon* (Bd21 and Bd3-1) in four genotype combinations and obtained only 48 seeds (Table 3). However, only 11 germinated and none of those were true interspecific hybrids (Table 3).

**Table 3:** Interspecific crosses made between autotetraploid (4x) plantsof *B*. *distachyon* and *B*. *stacei*.

♂♀	*B*. *stacei* 4×ABR114	*B*. *stacei* 4×Bsta5	*B*. *distachyon*4×Bd21	*B*. *distachyon*4×Bd3-1
***B*. *stacei* 4×ABR114**	_*	_	748^a^	550
			9^b^	4
			2^c^	1
			0^d^	0
***B*. *stacei*4×Bsta5**	_	_	469	484
			6	5
			2	1
			0	0
***B*. *distachyon*4×Bd21**	608	540	_	_
	12	2		
	4	0		
	0	0		
***B*. *distachyon*Bd3-1**	509	476	_	_
	7	3		
	2	0		
	0	0		

* No crosses made between lines of the same species

^a^ number of made crosses

^b^ number of seeds produced

^c^ number of seeds that germinated

^d^ number of true F1 interspecific hybrids based on molecular markers

### Phenotypic characterization of synthetic allotetraploids

**Fig 6:**
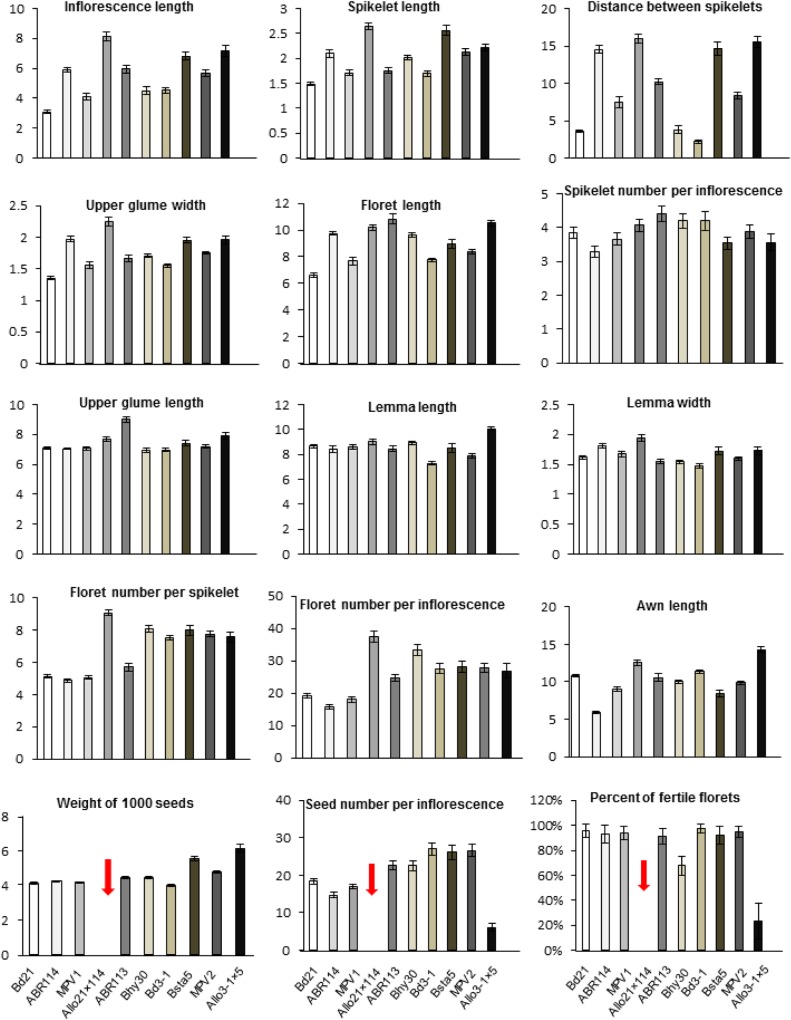
Comparison of spike and floret characters measured in synthetic allotetraploids (S1 generation), *B*. *distachyon*, *B*. *stacei* and natural *B*. *hybridum*. MPV1 = Mid-parent value: average of *B*. *distachyon* Bd21 and *B*. stacei ABR114 parent values; MPV2: average of *B*. *distachyon* Bd3-1 and *B*. *stacei* Bsta5 parent values. See S2 Fig for the specific characters scored and S3 Table for further details. There were no seeds from S1 plants of allo21×114, therefore seed number per inflorescence and percent of fertile florets were not scored.

Fifteen morphological characteristics of inflorescences, flowers and seeds were measured and compared between S1 generation allotetraploids, *B*. *distachyon*, *B*. *stacei* and *B*. *hybridum* (Fig 6; S3 Table). In general, the synthetic allotetraploids were more similar to natural *B*. *hybridum* and usually exceeded the parental species. More comparisons for each of the individual traits are detailed in S1 Text.

### Karyotype characterization

**Fig 7:**
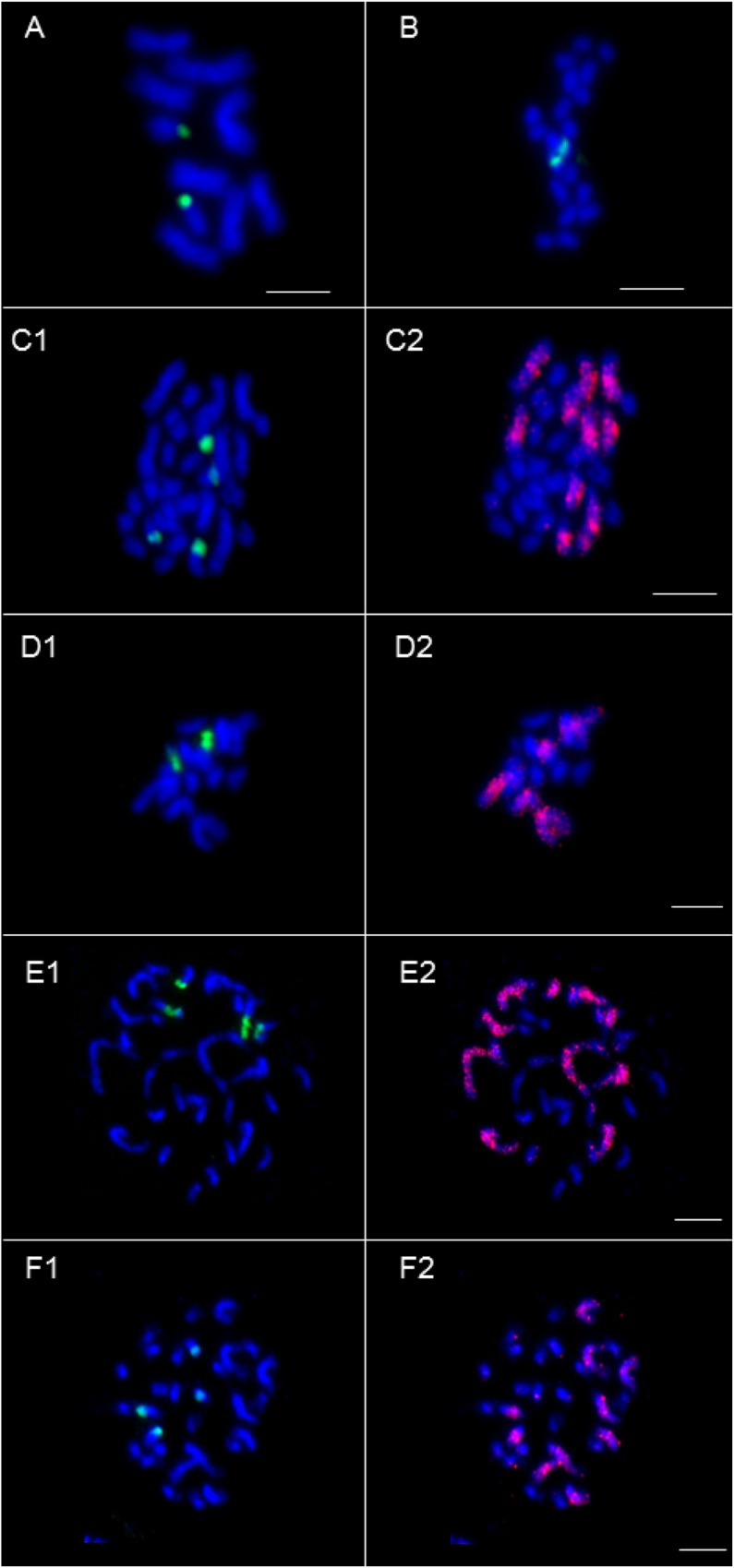
Cytogenetic analysis on metaphase chromosomes of natural *Brachypodium* species, F1 interspecific hybrid, and synthetic allotetraploids (Blue: DAPI staining, green: FISH with 45S rDNA probe; red: genome-specific discrimination of chromosomes with BAC clone ABR1-63-E6). (A-B) DAPI staining revealing five pairs of chromosomes in *B*. *distachyon* Bd21 (2n = 10) and 10 smaller chromosome pairs in *B*. *stacei* ABR114 (2n = 20) whereas FISH with 45S rDNA probe (green) reveals two sites in *B*. *distachyon* and two in *B*. *stacei*. (C) DAPI staining revealing 15 pairs of chromosomes in natural *B*. *hybridum* ABR113 (2n = 30) allotetraploid: five large chromosome pairs derived from the *B*. *distachyon* and 10 smaller pairs derived from the *B*. *stacei* parent. FISH with 45S rDNA probe (green) reveals four sites (two on each parent-derived chromosomes) (C1) and genome-specific discrimination of chromosomes with BAC clone ABR1-63-E6 probe (red) reveals specifically five large *B*. *distachyon*-derived chromosomes pairs and 10 smaller pairs (blue) derived from the *B*. *stacei* parent (C2). (D) F1 Interspecific amphihaploid hybrid (F1_3–1×5). DAPI staining (blue) revealing 5 *B*. *distachyon*-derived chromosomes and 10 smaller *B*. *stacei*-derived chromosomes (D1). FISH with 45S rDNA probe (green) reveals one site on a *B*. *distachyon*-derived chromosome and one other on a *B*. *stacei*-derived smaller chromosome (D1). Genome-specific discrimination of chromosomes with BAC clone ABR1-63-E6 probe reveals specifically five *B*. *distachyon*-derived chromosomes and 10 smaller (blue) *B*. *stacei*-derived chromosomes (D2). (E-F) S1 and S2 plants of the synthetic allotetraploid allo3-1×5 with DAPI staining and FISH (blue) with 45S rDNA probe (green) (E1, F1) and GISH-like with BAC clone ABR1-63-E6 (red, E2, F2), showing similar profiles to those of natural *B*. *hybridum*. Bars: 5 μm.

Metaphase chromosomal analysis was done in F1 interspecific amphihaploid hybrids and in S1 and S2 generation plants of the synthetic allotetraploid (Fig 7; Table 2). The comparative analysis showed the expected 10 large chromosomes in *B*. *distachyon* (Fig 7A), 20 small chromosomes in *B*. *stacei* (Fig 7B) and 30 (large and small) chromosomes in the natural allotetraploid *B*. *hybridum* (Fig 7C). Amphihaploid F1 interspecific hybrids contained 15 chromosomes, five derived from *B*. *distachyon* and 10 from *B*. *stacei*, (Fig 7D). As expected, chromosomes were duplicated in the two derived S1, S2 plants of the synthetic allopolyploid allo3-1×5 (Fig 7E and 7F) that had similar karyotypes to those of natural *B*. *hybridum*. FISH with the 45S rDNA probe labelled the expected number of spots in all lines: two spots in *B*. *distachyon*, *B*. *stacei* and their F1 amphihaploid hybrids (Fig 7A, 7B and 7D) and four spots in the S1 and S2 allotetraploid plants (Fig 7E and 7F) and natural *B*. *hybridum* (Fig 7C). Genome-specific chromosome discrimination with the BAC ABR1-63-E6 probe demonstrated the presence of five chromosomes from *B*. *distach*yon in the amphihaploid F1 interspecific hybrids (Fig 7D) and a doubled number (10) in their derived S1 and S2 synthetic allotetraploids (Fig 7E and 7F) and in the natural *B*. *hybridum* (Fig 7C). We analyzed karyotype of 53 cells from 10 different S2 allo3-1×5 plants.All karyotypes had the expected number of 30 chromosomes indicating that chromosomes are stably inherited (Table 2).

### Genetic characterization of synthetic allopolyploids

**Fig 8:**
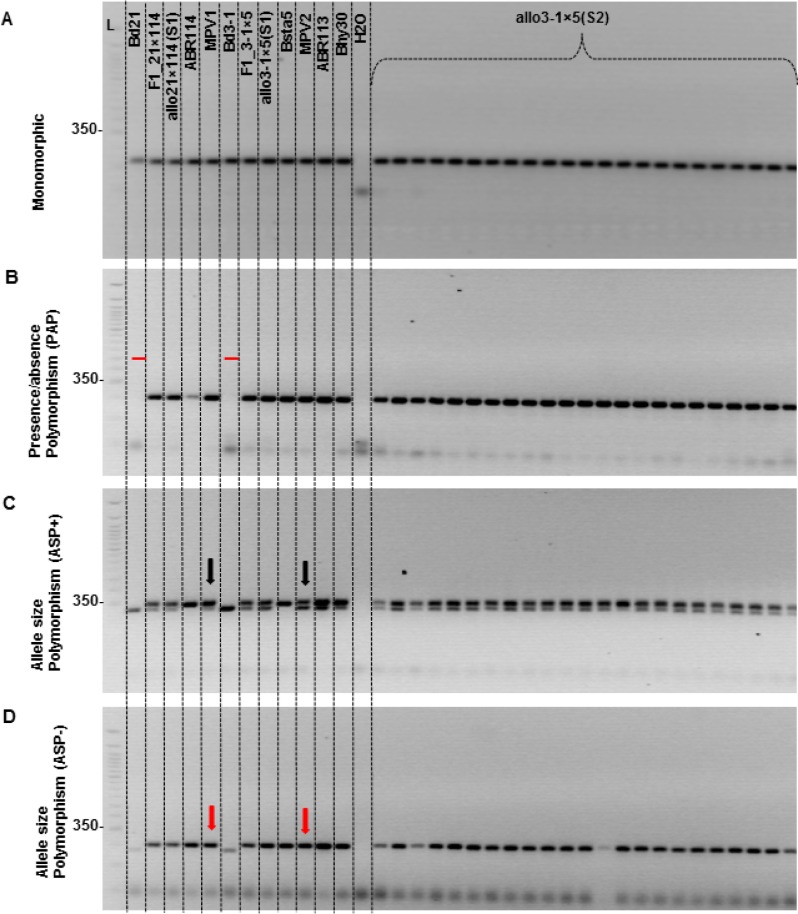
PCR-based marker profiles of plants from *B*. *distachyon*, *B*. *stacei*, their derived F1 interspecific hybrids and synthetic *B*. *hybridum (*S1 and S2 generations) as well as natural *B*. *hybridum*, and mixtures of equal amounts of parental DNAs (MPV1 and MPV2). (A) Marker Bd2-28 showing no polymorphism between *B*. *distachyon* (Bd21 and Bd3-1) and *B*. *stacei* (ABR114 and Bsta5) parent lines and no change in F1, S1 and S2 synthetic allopolyploid. (B) Marker Bd3-11 showing a presence⁄absence polymorphism (PAP) with a band in *B*. *stacei* and no band in *B*. *distachyon*. All F1, S1 and S2 plants have a band that is the same size as the *B*. *stacei* band. (C) Marker Bd5-14 exhibits allele size polymorphism (ASP+) between *B*. *distachyon* and *B*. *stacei*, with both parental alleles amplified in MPVs, F1, S1, S2, and natural *B*. *hybridum*. (D) Marker Bd1-26 exhibiting allele size polymorphism (ASP-) between *B*. *distachyon* (Bd21 and Bd3-1) and *B*. *stacei* (ABR114 and Bsta5). In this case, while the parents have different sized bands, the *B*. *distachyon* allele is not amplified in MPVs (red arrows), natural *B*. *hybridum* or the F1, S1 and S2 generations. Absence of the *B*. *distachyon* allele is most likely due to competition for PCR amplification between progenitor alleles and not from DNA rearrangements. L: 50 bp ladder (Invitrogen, Carlsbad, CA, USA). MPV1; Mixture of DNA from Bd21 and ABR114. MPV2: Mixture of DNA from Bd3-1 and Bsta5.

SSR- and gene-derived PCR markers were used to characterize the genetic stability of synthetic allotetraploids. The single allo3-1×5 S1 plant was fertile, allowing us to examine 118 individual S2 progeny, whereas only a single sterile allo21×114 S1 plant was analyzed. The genetic markers were classified based on the polymorphism observed between parental species, their pooled DNAs, and the natural allotetraploid *B*. *hybridum* using the momenclature recommended in [43]. The polymorphism pattern observed in the synthetic allotetraploids was identical to natural *B*. *hybridum* and was the same for all S2 individuals examined, as illustrated in Fig 8 and described in more details in S2 Text

A total of 151 markers (129 gene-based and 22 SSR markers) and 140 markers (123 gene-based and 17 SSR markers) were analyzed for allo21×114 and allo3-1×5, respectively. The amplification patterns observed for F1 interspecific amphihaploid hybrids, F1_21×114 and F1_3–1×5, were the same as those observed for their derived S0 and S1 synthetic allopolyploids (Table 4).

**Table 4:** Classification of gene- and simple sequence repeats (SSRs)-based PCR markers according to their amplification patterns observed on *B*. *distachyon* and *B*. *stacei* diploid species, their pooled DNAs (MPVs), their F1 interspecific hybrids and S0, S1 and S2 generations of the derived synthetic allotetraploids.

Marker classification	Genome	Chr	M	PAP	ASP ^a^	Total
**Gene based markers**	Allo21×114	Bd1	2	14	11 (4)	**27**
		Bd2	4	12	8 (1)	**24**
		Bd3	0	14	10 (1)	**24**
		Bd4	12	7	10 (2)	**29**
		Bd5	3	14	8 (2)	**25**
		**Total**	**21**	**61**	**47**	**129**
	Allo3-1×5	Bd1	4	11	11(4)	**26**
		Bd2	5	10	8 (1)	**23**
		Bd3	0	9	11 (1)	**20**
		Bd4	14	5	10 (2)	**29**
		Bd5	4	13	8 (2)	**25**
		**Total**	**27**	**48**	**48**	**123**
**SSR markers**	Allo21×114	Bd1	0	3	1	**4**
		Bd2	0	6	1	**7**
		Bd3	0	1	1(1)	**3**
		Bd4	1	2	1(1)	**5**
		Bd5	0	1	1(1)	**3**
		**Total**	**1**	**13**	**8**	**22**
	Allo3-1×5	Bd1	1	2	1	**3**
		Bd2	0	4	1	**5**
		Bd3	0	1	2	**3**
		Bd4	1	2	1	**4**
		Bd5	0	0	1(1)	**2**
		**Total**	**1**	**9**	**7**	**17**

Chr: Chromosome number according to *B*. *distachyon* (Bd)

M: monomorphic

PAP: presence/absence polymorphic markers, amplifying one single parental allele

ASP: allele specific markers, amplifying both parental alleles with polymorphic allelic size differences. Numbers between brackets correspond to those that amplify only one parental allele in pooled DNA from both parents.

No evidence of DNA rearrangements in the F1 interspecific amphihaploid hybrids or the derived synthetic S0 and S1 plants was found (Fig 8; Table 4). Similarly, none of the 118 analyzed S2 allo3-1×5 plants showed rearrangements of parental alleles (Fig 8; Table 4), indicating genomic stability.

The genetic patterns in the synthetic allotetraploids were almost identical to the genetic profiles observed in natural *B*. *hybridum* ABR113 and Bhyb30 lines (S2 Table). However, consistent with the parental genotypes, we observed slightly more differences with line Bhyb30 than with line ABR113.

## Discussion

The origin and evolutionary relationships of the natural *B*. *hybridum* allotetraploid in relation to its progenitor species *B*. *distachyon* and *B*. *stacei* is now clearly elucidated [26, 32–34, 69]. The creation of synthetic allotetraploid plants similar to natural *B*.*hybridum* provides empirical evidence and establishes the tractable *Brachypodium* allotetraploid model. This represents a unique allopolyploid model where one parental genome (*B*. *distachyon*) has similar genome size to the other one (*B*. *stacei*), but half the sporophytic (2n) chromosome number (2n = 10 and 2n = 20, respectively) whereas its individual chromosome size is approximately two times larger.

The existence of *B*. *hybridum* and other natural *Brachypodium* allopolyploids [24] as well as our success in synthesizing *B*. *hybridum*–type allotetraploids, suggest that differences in chromosome size and number between progenitor species do not constitute a barrier to interspecific hybridization and allopolyploid formation. It has been suggested that *B*. *distachyon* and its derived allotetraploid *B*. *hybridum* have hybridized with various other diploid species [24, 70], presumably leading to several additional *Brachypodium* allopolyploids including *B*. *pinnatum* (2n = 28), *B*. *phoenicoides* (2n = 28), *B*. *phoenicoides* (2n = 28) and *B*. *retusum* (2n = 38) [30, 31, 33, 71].

Moreover, the *B*. *hybridum*-type allotetraploids synthesized here appear highly stable from the earliest generations (S1 and S2) as characterized at the phenotypic, cytogenetic and genetic levels. The prominent differences in chromosome number and chromosome size of the two parental genomes likely serve as a barrier to homoeologous pairing which may contribute to the chromosomal stability of both natural and synthetic *B*. *hybridum*. We will investigate this further by studying meiosis and chromosome pairing in these model allotetraploids.

Evidence suggests that *B*. *hybridum* allotetraploids formed naturally more than once with both *B*. *distachyon* and *B*. *stacei* as the maternal parent [34]. In our experiments, all surviving synthetic allotetraploids had *B*. *distachyon* as the maternal parent and no allopolyploids were obtained from reciprocal crosses. Moreover, the success rate of interspecific hybridization in the present study was very low. This is similar to other species where parental genotypes have been shown to have a large effect on the success of interspecific hybridization [72–74].

It has been suggested that a combination of factors, including differences in flowering time, pollinator behavior and floral structure, caused by both biological and genetic factors, limit hybridization between distantly-related species [75]. Even when pollination occurs, post-pollination barriers, such as differences in style structure and the arrest of the pollen tube growth, can inhibit the formation of zygotes between different species. These can be overcome by refining crossing methods [76]. As an example, in lilies (*Lilium candidum* L.) the pollen tubes arrest halfway down the style after interspecific pollination, a barrier that can be overcome by *in vitro* methods [77]. Post-pollination barriers have also been reported in species, such as *Rhinanthus* and *Nicotiana* [78, 79], as the pollen tube developement at different rate in hetero-specific style or because of differences in pistil length between the crossed species.

In the present study, by performing a high number of interspecific crosses, we obtained viable F1 interspecific hybrids in only two out of four genotype combinations. In comparison, intraspecific hybridizations between divergent lines of each of the two parental species (including the ones used in this study) could be realized relatively easily with a high success rate (http://jgi.doe.gov/our-science/science-programs/plant-genomics/brachypodium/). Previously, other groups have failed to obtain F1 interspecific amphihaploid hybrids between other *Brachypodium* species [70]. Significantly, the successful combination of lines Bsta5 of *B*. *stacei* and Bd3-1 of *B*. *distachyon* was not tried in previous attemps (G. Linc and R. Hasterok, unpublished). This illustrates the importance of the parental genotypes and the need to conduct a very large number of crosses. Further characterization of the germination of the pollen on the stigma papilla as well as the progression of the pollen tubes in the style may elucidate the barriers limiting zygote and interspecific hybrid formation between *B*. *distachyon* and *B*. *stacei*.

Interspecific F1 amphihaploid hybrids are normally sterile because the parental chromosomes do not pair normally during meiosis leading to unbalanced non-viable gametes [80, 81]. Doubling the genome of F1 amphihaploid plants often restores fertility and occasionally this occurs spontaneously as has been observed in a variety of plant species such as in wheat [43], *Arabidopsis* [64, 82–84], and rice [85]. In our study, no seeds were obtained from thousands of amphihaploid F1 interspecific hybrid flowers, indicating that restoration by spontaneous genome doubling does not occur or is exceedingly rare for the crosses we made. This would suggest that *B*. *hybridum* may have been formed naturally by hybridization between unreduced gametes that are rarely produced by the diploid parents. Nevertheless, crosses between the autotetraploid lines of *B*. *distachyon* and *B*. *stacei*, that produce 2n gametes, were not also successful. A possible reason for the failure of the autotetraploid crosses is the low fertility of the *B*. *stacei* and *B*. *distachyon* autotetraploid lines (46% and 82%, respectively) and reduced pollen viability (data not shown). We were able to artificially double the genome of our two amphihaploid F1 hybrid plants leading to low fertility. Interestingly, for one cross, the next selfed generation (S1) was even more fertile. It will be very interesting to explore the changes responsible for such increasing fertility. Conversely, fertility did not increased in the S1 generation of the other allopolyploid and this contrast may provide mechanistic insight. Whilst reasons of sterility of allo21×114 in comparison to the fertile allo3-1×5 allotetraploid need to be investigated, these findings suggests the existence of pre-established genetic or structural fertility barriers that influence hybridization success and stability of allopolyploid genomes, as observed for hexaploid wheat [43].

In conclusion, the successful synthesis of allotetraploids similar to the natural *B*. *hybridum* provides a powerful new tool to an emerging polyploid model system. When combined with the experimental resources and experimental tractability of *B*. *distachyon*, *B*. *stacei* and *B*. *hybridum*, the ability to create allotetraploids opens up exciting possibilities to study various aspects of polyploidy in grasses at genomic, cytomolecular, epigenetic and physiological levels from the very earliest stages of their formation.

## Supporting Information

S1 Fig Vegetative propagation of F1 amphihaploid hybrids, S0 and S1 generations of *Brachypodium* synthetic allopolyploids. (A) Tillers with secondary roots emerging from the node (indicated by arrows) were used for propagation. (B) Stimulation rooting by burying tiller node in the soil containing 0.25% indole-3-butyric Acid (IBA). The tiller was held in place with a stick. (C) Rooted tillers cut from the initial plant: (1) A tiller with enough roots to live independently, (2) A tiller with no roots. (D) The rooted tiller was transferred directly into a pot to produce a new plant. (E) The rootless tiller was placed in water containing 0.25% IBA. After 7–10 days, the root emerged (indicated by blue arrows) and grew enough to transfer this tiller into a new pot.
(TIF)

S2 Fig Phenotypic characters recorded in the present study. 1. Inflorescence length; 2. Spikelet length; 3. Distance between spikelets; 4. Upper glume from basal spikelet length; 5. Upper glume from basal spikelet width; 6. floret length (the second floret of all spikelets on one inflorescence were measured); 7. Awn length; 8. Lemma length; 9. Lemma width. Other characters measured for inflorescence or spikelet: 10. Spikelet number per inflorescence (all spikelets in the spike—5 in this example); 11. Floret number per spikelet (i.e. number of florets in each spikelet); 12. Floret number per inflorescence (i.e. all florets in an inflorescence); 13. Seed number per inflorescence; 14. Percentage of fertile florets; 15. Weight of 1,000
(TIF)

S3 Fig Male sterility in the F1 interspecific hybrid F1_21×114 and S1 generation of its derived synthetic allopolyploid allo21×114, compared to diploid progenitors *B*. *distachyon (*Bd21) and *B*. *stacei (*ABR114) and the natural allopolyploid *B*. *hybridum* (ABR113). (A) Anthers on the day of anthesis. (B) Spontaneous release of the pollen from anthers after 15–20 minutes incubation on a microscope slide: (B) Bd21, (C) ABR113 and (D) ABR114. This phenomenon was not observed for (E) F1_21×114 and (F) S1 generation of allo21×114; after macerating these anthers there was very little pollen compared with progenitors and natural polyploid.
(TIF)

S1 Table Results of the vegetative multiplication of amphihaploid F1 interspecific hybrids and their treatment with varying concentrations of colchicine (2.5 g/l, 5g/l and 7.5 g/l). (DOCX)

S2 Table Primer sequences and characteristics of PCR-based polymorphic markers derived from gene and SSR sequences. (XLSX)

S3 Table Mean values ± standard deviation for 15 morphological traits measured in different synthetic and natural *Brachypodium* allopolyploids and their parental species. (XLSX)

S4 Table Floal characters of *B*. *distachyon*, *B*. *stacei*, *B*. *hybridum*, interspecific F1 hybrids and plants of S1 generation of the synthetic allopolyploids allo21×114 and allo3-1×5. (DOCX)

S5 Table Number of crosses made between the diploid parental species *B*. *distachyon* and *B*. *stacei* as well as *B*. *hybridum* natural allopolyploids with the interspecific hybrid F1_21×114 and the synthetic allotetraploid allo21×114(S1 generation). (DOCX)

S1 Text Phenotypic characterization of synthetic allopolyploids. (DOCX)

S2 Text Classification and interpretation of marker polymorphism (DOCX)

## References

[1] Barker MS, Arrigo N, Baniaga AE, Li Z, Levin DA. On the relative abundance of autopolyploids and allopolyploids. New Phytol. 2016;210(2):391–8. Epub 2015/10/07. 10.1111/nph.13698 26439879

[2] Blanc G, Wolfe KH. Widespread Paleopolyploidy in Model Plant Species Inferred from Age Distributions of Duplicate Genes. The Plant Cell. 2004;16(7):1667–78. 10.1105/tpc.021345 15208399 PMC514152

[3] Adams KL, Wendel JF. Polyploidy and genome evolution in plants. Curr Opin Plant Biol. 2005;8(2):135–41. Epub 2005/03/09. 10.1016/j.pbi.2005.01.001 15752992

[4] Tang H, Bowers JE, Wang X, Ming R, Alam M, Paterson AH. Synteny and collinearity in plant genomes. Science. 2008;320(5875):486–8. Epub 2008/04/26. 10.1126/science.1153917 18436778

[5] Barker MS, Baute GJ, Liu S-L. Duplications and Turnover in Plant Genomes In: Wendel FJ, Greilhuber J, Dolezel J, Leitch JI, editors. Plant Genome Diversity Volume 1: Plant Genomes, their Residents, and their Evolutionary Dynamics. Vienna: Springer Vienna; 2012 p. 155–69.

[6] Van de Peer Y, Maere S, Meyer A. The evolutionary significance of ancient genome duplications. Nat Rev Genet. 2009;10(10):725–32. 10.1038/nrg2600 19652647

[7] Wendel JF, Jackson SA, Meyers BC, Wing RA. Evolution of plant genome architecture. Genome biology. 2016;17(1):37. Epub 2016/03/02. PubMed Central PMCID: PMCPMC4772531. 26926526 10.1186/s13059-016-0908-1 PMC4772531

[8] Beest M, Le Roux JJ, Richardson DM, Brysting AK, Suda J, Kubešová M, et al The more the better? The role of polyploidy in facilitating plant invasions. Annals of Botany. 2011;109(1):19–45. 10.1093/aob/mcr277 22040744 PMC3241594

[9] Buggs RJ, Renny-Byfield S, Chester M, Jordon-Thaden IE, Viccini LF, Chamala S, et al Next-generation sequencing and genome evolution in allopolyploids. Am J Bot. 2012;99(2):372–82. Epub 2012/01/24. 10.3732/ajb.1100395 22268220

[10] Chalhoub B, Denoeud F, Liu S, et al Plant genetics. Early allopolyploid evolution in the post-Neolithic *Brassica napus* oilseed genome. Science. 2014;345(6199):950–3. 10.1126/science.1253435 25146293

[11] Ghani MA, Li J, Rao L, Raza MA, Cao L, Yu N, et al The role of small RNAs in wide hybridisation and allopolyploidisation between *Brassica rapa* and *Brassica nigra*. BMC Plant Biol. 2014;14:272 Epub 2014/10/20. PubMed Central PMCID: PMCPMC4209033. 10.1186/s12870-014-0272-9 25326708 PMC4209033

[12] Kashkush K, Feldman M, Levy AA. Transcriptional activation of retrotransposons alters the expression of adjacent genes in wheat. Nature Genetics. 2003;33(1):102–6. 10.1038/ng1063 12483211

[13] Rapp RA, Udall JA, Wendel JF. Genomic expression dominance in allopolyploids. BMC biology. 2009;7:18 Epub 2009/05/05. PubMed Central PMCID: PMCPMC2684529. 10.1186/1741-7007-7-18 19409075 PMC2684529

[14] Rieseberg LH, Sinervo B, Linder CR, Ungerer MC, Arias DM. Role of gene interactions in hybrid speciation: evidence from ancient and experimental hybrids. Science. 1996;272(5262):741–5. Epub 1996/05/03. 8662570 10.1126/science.272.5262.741

[15] Song K, Lu P, Tang K, Osborn TC. Rapid genome change in synthetic polyploids of *Brassica* and its implications for polyploid evolution. Proceedings of the National Academy of Sciences of the United States of America. 1995;92(17):7719–23. Epub 1995/08/15. PubMed Central PMCID: PMCPMC41217. 7644483 10.1073/pnas.92.17.7719 PMC41217

[16] Stebbins GL. Polyploidy, Hybridization, and the Invasion of New Habitats. Annals of the Missouri Botanical Garden. 1985;72(4):824–32.

[17] Paun O, Bateman RM, Fay MF, Luna JA, Moat J, Hedren M, et al Altered gene expression and ecological divergence in sibling allopolyploids of *Dactylorhiza* (*Orchidaceae*). BMC evolutionary biology. 2011;11:113 Epub 2011/04/28. PubMed Central PMCID: PMCPMC3112086. 10.1186/1471-2148-11-113 21521507 PMC3112086

[18] Estep MC, McKain MR, Vela Diaz D, Zhong J, Hodge JG, Hodkinson TR, et al Allopolyploidy, diversification, and the Miocene grassland expansion. Proceedings of the National Academy of Sciences. 2014;111(42):15149–54. 10.1073/pnas.1404177111 PMC4210326 25288748

[19] Soreng RJ, Peterson PM, Romaschenko K, Davidse G, Zuloaga FO, Judziewicz EJ, et al A worldwide phylogenetic classification of the Poaceae (Gramineae). Journal of Systematics and Evolution. 2015;53(2):117–37.

[20] Kellogg EA. Evolutionary history of the grasses. Plant Physiol. 2001;125(3):1198–205. Epub 2001/03/13. PubMed Central PMCID: PMCPMC1539375. 11244101 10.1104/pp.125.3.1198 PMC1539375

[21] Bouchenak-Khelladi Y, Slingsby JA, Verboom GA, Bond WJ. Diversification of C4 grasses (Poaceae) does not coincide with their ecological dominance. American Journal of Botany. 2014;101(2):300–7. 10.3732/ajb.1300439 24509796

[22] Chalupska D, Lee HY, Faris JD, Evrard A, Chalhoub B, Haselkorn R, et al Acc homoeoloci and the evolution of wheat genomes. Proceedings of the National Academy of Sciences. 2008;105(28):9691–6. 10.1073/pnas.0803981105 PMC2474508 18599450

[23] Draper J, Mur LA, Jenkins G, Ghosh-Biswas GC, Bablak P, Hasterok R, et al *Brachypodium distachyon*. A new model system for functional genomics in grasses. Plant Physiol. 2001;127(4):1539–55. PubMed Central PMCID: PMC133562. 11743099 PMC133562

[24] Catalan P, López-Álvarez D, Díaz-Pérez A, Sancho R, López-Herránz ML. Phylogeny and Evolution of the Genus Brachypodium In: Vogel PJ, editor. Genetics and Genomics of Brachypodium. Cham: Springer International Publishing; 2016 p. 9–38.

[25] Catalan P, Kellogg EA, Olmstead RG. Phylogeny of Poaceae subfamily Pooideae based on chloroplast ndhF gene sequences. Mol Phylogenet Evol. 1997;8(2):150–66. Epub 1997/09/23. 10.1006/mpev.1997.0416 9299221

[26] Catalan P, Chalhoub B, Chochois V, Garvin DF, Hasterok R, Manzaneda AJ, et al Update on the genomics and basic biology of *Brachypodium*: International *Brachypodium* Initiative (IBI). Trends Plant Sci. 2014;19(7):414–8. 10.1016/j.tplants.2014.05.002 24917149

[27] Brkljacic J, Grotewold E, Scholl R, Mockler T, Garvin DF, Vain P, et al *Brachypodium* as a model for the grasses: today and the future. Plant Physiol. 2011;157(1):3–13. Epub 2011/07/21. PubMed Central PMCID: PMCPMC3165879. 10.1104/pp.111.179531 21771916 PMC3165879

[28] IBI T. Genome sequencing and analysis of the model grass *Brachypodium distachyon*. Nature. 2010;463(7282):763–8. Epub 2010/02/12. 10.1038/nature08747 20148030

[29] Robertson IH. Chromosome numbers in *Brachypodium* Beauv. (Gramineae). Genetica. 1981;56(1):55–60.

[30] Wolny E, Hasterok R. Comparative cytogenetic analysis of the genomes of the model grass *Brachypodium distachyon* and its close relatives. Ann Bot. 2009;104(5):873–81. Epub 2009/07/28. PubMed Central PMCID: PMCPMC2749528. 10.1093/aob/mcp179 19633311 PMC2749528

[31] Betekhtin A, Jenkins G, Hasterok R. Reconstructing the Evolution of *Brachypodium* Genomes Using Comparative Chromosome Painting. PLoS One. 2014;9(12):e115108 PubMed Central PMCID: PMCPMC4262448. 10.1371/journal.pone.0115108 25493646 PMC4262448

[32] Hasterok R, Draper J, Jenkins G. Laying the cytotaxonomic foundations of a new model grass, *Brachypodium distachyon* (L.) Beauv. Chromosome Res. 2004;12(4):397–403. Epub 2004/07/09. 10.1023/B:CHRO.0000034130.35983.99 15241018

[33] Catalan P, Muller J, Hasterok R, Jenkins G, Mur LA, Langdon T, et al Evolution and taxonomic split of the model grass *Brachypodium distachyon*. Ann Bot. 2012;109(2):385–405. Epub 2012/01/04. PubMed Central PMCID: PMCPMC3268539. 10.1093/aob/mcr294 22213013 PMC3268539

[34] Lopez-Alvarez D, Lopez-Herranz ML, Betekhtin A, Catalan P. A DNA barcoding method to discriminate between the model plant *Brachypodium distachyon* and its close relatives *B*. *stacei* and *B*. *hybridum* (Poaceae). PLoS One. 2012;7(12):e51058 Epub 2012/12/15. PubMed Central PMCID: PMCPMC3519806. 10.1371/journal.pone.0051058 23240000 PMC3519806

[35] Gordon SP, Liu L, Vogel JP. The Genus Brachypodium as a Model for Perenniality and Polyploidy In: Vogel PJ, editor. Genetics and Genomics of Brachypodium. Cham: Springer International Publishing; 2016 p. 313–25.

[36] Mason AS, Pires JC. Unreduced gametes: meiotic mishap or evolutionary mechanism? Trends in Genetics. 2015;31(1):5–10. 10.1016/j.tig.2014.09.011 25445549

[37] Ramsey J, Schemske DW. Pathways, mechanisms, and rates of polyploid formation in flowering plants. Annual Review of Ecology and Systematics. 1998;29:467–501.

[38] Osabe K, Kawanabe T, Sasaki T, Ishikawa R, Okazaki K, Dennis ES, et al Multiple mechanisms and challenges for the application of allopolyploidy in plants. International journal of molecular sciences. 2012;13(7):8696–721. Epub 2012/09/04. PubMed Central PMCID: PMCPmc3430260. 10.3390/ijms13078696 22942729 PMC3430260

[39] Chen ZJ, Ni Z. Mechanisms of genomic rearrangements and gene expression changes in plant polyploids. BioEssays: news and reviews in molecular, cellular and developmental biology. 2006;28(3):240–52. Epub 2006/02/16. PubMed Central PMCID: PMCPmc1986666. 10.1002/bies.20374 PMC1986666 16479580

[40] Szadkowski E, Eber F, Huteau V, Lode M, Coriton O, Jenczewski E, et al Polyploid formation pathways have an impact on genetic rearrangements in resynthesized *Brassica napus*. New Phytol. 2011;191(3):884–94. 10.1111/j.1469-8137.2011.03729.x 21517871

[41] Madlung A, Masuelli RW, Watson B, Reynolds SH, Davison J, Comai L. Remodeling of DNA Methylation and Phenotypic and Transcriptional Changes in Synthetic *Arabidopsis* Allotetraploids. Plant Physiology. 2002;129(2):733–46. 10.1104/pp.003095 12068115 PMC161697

[42] Lim KY, Souckova-Skalicka K, Sarasan V, Clarkson JJ, Chase MW, Kovarik A, et al A genetic appraisal of a new synthetic *Nicotiana tabacum* (*Solanaceae*) and the Kostoff synthetic tobacco. Am J Bot. 2006;93(6):875–83. Epub 2006/06/01. 10.3732/ajb.93.6.875 21642150

[43] Mestiri I, Chagué V, Tanguy AM, Huneau C, Huteau V, Belcram H, et al Newly synthesized wheat allohexaploids display progenitor-dependent meiotic stability and aneuploidy but structural genomic additivity. New Phytol. 2010;186(1):86–101. 10.1111/j.1469-8137.2010.03186.x 20149116

[44] Ng DW, Zhang C, Miller M, Shen Z, Briggs SP, Chen ZJ. Proteomic divergence in *Arabidopsis* autopolyploids and allopolyploids and their progenitors. Heredity (Edinb). 2012;108(4):419–30. PubMed Central PMCID: PMC3313054. 22009271 10.1038/hdy.2011.92 PMC3313054

[45] Comai L. Genetic and epigenetic interactions in allopolyploid plants. Plant Mol Biol. 2000;43(2–3):387–99. 10999418 10.1023/a:1006480722854

[46] Madlung A, Wendel JF. Genetic and epigenetic aspects of polyploid evolution in plants. Cytogenetic and genome research. 2013;140(2–4):270–85. 10.1159/000351430 23751292

[47] Vogel JP, Garvin DF, Leong OM, Hayden DM. Agrobacterium-mediated transformation and inbred line development in the model grass Brachypodium distachyon. Plant Cell, Tissue and Organ Culture. 2006;84(2):199–211.

[48] Ludwig-Muller J, Vertocnik A, Town CD. Analysis of indole-3-butyric acid-induced adventitious root formation on *Arabidopsis* stem segments. J Exp Bot. 2005;56(418):2095–105. Epub 2005/06/16. 10.1093/jxb/eri208 15955788

[49] Jahier J. Techniques of plant cytogenetics. INRA Edition ed: INRA Edition, Paris, France; 1992.

[50] Cousin A, Heel K, Cowling WA, Nelson MN. An efficient high-throughput flow cytometric method for estimating DNA ploidy level in plants. Cytometry Part A. 2009;75A(12):1015–9. 10.1002/cyto.a.20816 19845019

[51] Besnard G, Garcia-Verdugo C, Rubio De Casas R, Treier UA, Galland N, Vargas P. Polyploidy in the Olive Complex (*Olea europaea*): Evidence from Flow Cytometry and Nuclear Microsatellite Analyses. Annals of Botany. 2008;101(1):25–30. 10.1093/aob/mcm275 18024415 PMC2701839

[52] Dart S, Kron P, Mable BK. Characterizing polyploidy in *Arabidopsis lyrata* using chromosome counts and flow cytometry. Canadian Journal of Botany. 2004;82(2):185–97.

[53] Suay L, Zhang D, Eber F, Jouy H, Lode M, Huteau V, et al Crossover rate between homologous chromosomes and interference are regulated by the addition of specific unpaired chromosomes in Brassica. New Phytol. 2014;201(2):645–56. Epub 2013/10/15. 10.1111/nph.12534 24117470

[54] Leflon M, Eber F, Letanneur JC, Chelysheva L, Coriton O, Huteau V, et al Pairing and recombination at meiosis of Brassica rapa (AA) x Brassica napus (AACC) hybrids. Theor Appl Genet. 2006;113(8):1467–80. Epub 2006/09/20. 10.1007/s00122-006-0393-0 16983552

[55] Hasterok R, Jenkins G, Langdon T, Jones RN. The nature and destiny of translocated B-chromosome-specific satellite DNA of rye. Chromosome Res. 2002;10(1):83–6. 11863075 10.1023/a:1014278429178

[56] Hasterok R, Dulawa J, Jenkins G, Leggett M, Langdon T. Multi-substrate chromosome preparations for high throughput comparative FISH. BMC biotechnology. 2006;6:20 PubMed Central PMCID: PMC1481663. 10.1186/1472-6750-6-20 16549000 PMC1481663

[57] Gerlach WL, Bedbrook JR. Cloning and characterization of ribosomal RNA genes in wheat. Nucleic Acids Research. 1979;7:1869–85. 537913 10.1093/nar/7.7.1869 PMC342353

[58] Charles M, Belcram H, Just J, Huneau C, Viollet A, Couloux A, et al Dynamics and differential proliferation of transposable elements during the evolution of the B and A genomes of wheat. Genetics. 2008;180(2):1071–86. Epub 2008/09/11. PubMed Central PMCID: PMCPMC2567357. 10.1534/genetics.108.092304 18780739 PMC2567357

[59] Giraldo P, Rodriguez-Quijano M, Vazquez JF, Carrillo JM, Benavente E. Validation of microsatellite markers for cytotype discrimination in the model grass *Brachypodium distachyon*. Genome. 2012;55(7):523–7. Epub 2012/07/14. 10.1139/g2012-039 22788413

[60] Garvin DF, McKenzie N, Vogel JP, Mockler TC, Blankenheim ZJ, Wright J, et al An SSR-based genetic linkage map of the model grass *Brachypodium distachyon*. Genome. 2010;53(1):1–13. Epub 2010/02/05. 10.1139/g09-079 20130744

[61] Hammami J, Soler, Frieiro, González. Genetic diversity of SSR and ISSR markers in wild populations of *Brachypodium distachyon* and its close relatives *B*. *stacei* and *B*. *hybridum* (Poaceae). Plant Systematics and Evolution. 2014; 300:2029–40.

[62] Vogel JP, Tuna M, Budak H, Huo N, Gu YQ, Steinwand MA. Development of SSR markers and analysis of diversity in Turkish populations of *Brachypodium distachyon*. BMC Plant Biol. 2009;9:88 Epub 2009/07/15. PubMed Central PMCID: PMCPMC2719641. 10.1186/1471-2229-9-88 19594938 PMC2719641

[63] Kruskal WH, Wallis WA. Use of Ranks in One-Criterion Variance Analysis. Journal of the American Statistical Association. 1952;47(260):583–621.

[64] Nasrallah ME, Yogeeswaran K, Snyder S, Nasrallah JB. *Arabidopsis* species hybrids in the study of species differences and evolution of amphiploidy in plants. Plant Physiol. 2000;124(4):1605–14. Epub 2000/12/15. PubMed Central PMCID: PMCPMC59859. 11115878 10.1104/pp.124.4.1605 PMC59859

[65] Choudhary BR, Joshi P, Ramarao S. Interspecific hybridization between *Brassica carinata* and *Brassica rapa*. Plant Breeding. 2000;119(5):417–20.

[66] Gangadevi T, Rao PN, Rao BH, Satyanarayana KV. A study of morphology, cytology and sterility in interspecific hybrids and amphidiploids of *Nicotiana knightiana* X N. *umbratica*. Theor Appl Genet. 1985;70(3):330–2. Epub 1985/06/01. 10.1007/BF00304921 24252931

[67] Poysa V. The development of bridge lines for interspecific gene transfer between *Lycopersicon esculentum* and *L*. *peruvianum*. Theor Appl Genet. 1990;79(2):187–92. Epub 1990/02/01. 10.1007/BF00225950 24226217

[68] Hong SY, Park JH, Cho SH, Yang MS, Park CM. Phenological growth stages of *Brachypodium distachyon*: codification and description. Weed Research. 2011;51(6):612–20.

[69] Idziak D, Hasterok R. Cytogenetic evidence of nucleolar dominance in allotetraploid species of *Brachypodium*. Genome. 2008;51(5):387–91. Epub 2008/04/29. 10.1139/G08-017 18438442

[70] Khan MA, Stace CA. Breeding relationships in the genus Brachypodium (Poaceae: Pooideae). Nordic Journal of Botany1999. p. 257–68.

[71] Idziak D, Hazuka I, Poliwczak B, Wiszynska A, Wolny E, Hasterok R. Insight into the karyotype evolution of *Brachypodium* species using comparative chromosome barcoding. PLoS One. 2014;9(3):e93503 Epub 2014/03/29. PubMed Central PMCID: PMCPMC3968144. 10.1371/journal.pone.0093503 24675822 PMC3968144

[72] Yao X, Ge X, Li Z. Different fertility and meiotic regularity in allohexaploids derived from trigenomic hybrids between three cultivated *Brassica* allotetraploids and *B*. *maurorum*. Plant Cell Rep. 2012;31(4):781–8. Epub 2011/12/08. 10.1007/s00299-011-1200-1 22147137

[73] Tate JA, Symonds VV, Doust AN, Buggs RJ, Mavrodiev E, Majure LC, et al Synthetic polyploids of Tragopogon miscellus and T. mirus (Asteraceae): 60 Years after Ownbey's discovery. Am J Bot. 2009;96(5):979–88. 10.3732/ajb.0800299 21628250

[74] Mejia-Jimenez A, Munoz C, Jacobsen HJ, Roca WM, Singh SP. Interspecific hybridization between common and tepary beans: increased hybrid embryo growth, fertility, and efficiency of hybridization through recurrent and congruity backcrossing. Theor Appl Genet. 1994;88(3–4):324–31. Epub 1994/06/01. 10.1007/BF00223640 24186014

[75] Presgraves DC, Glor RE. Evolutionary biology: speciation on islands. Current biology: CB. 2010;20(10):R440–2. 10.1016/j.cub.2010.03.032 20504751

[76] Sitch LA, Snape JW. Factors affecting haploid production in wheat using the *Hordeum bulbosum* system. 3. Post-fertilization effects on embryo survival. Euphytica. 1987;36(2):497–504.

[77] Taylor NL, Quarles RF, Anderson MK. Methods of overcoming interspecific barriers in *Trifolium*. Euphytica. 1980;29(2):441–50.

[78] Lee C, Page L, McClure B, Holtsford T. Post-pollination hybridization barriers in *Nicotiana* section Alatae. Sex Plant Reprod. 2008;21(3):183–95.

[79] Natalis LC, Wesselingh RA. Post-pollination barriers and their role in asymmetric hybridization in *Rhinanthus* (Orobanchaceae). American Journal of Botany. 2012;99(11):1847–56. 10.3732/ajb.1200085 23092992

[80] Scopece G, Widmer A, Cozzolino S. Evolution of postzygotic reproductive isolation in a guild of deceptive orchids. The American naturalist. 2008;171(3):315–26. Epub 2008/01/18. 10.1086/527501 18198999

[81] Ouyang Y, Zhang Q. Understanding reproductive isolation based on the rice model. Annu Rev Plant Biol. 2013;64:111–35. Epub 2013/05/04. 10.1146/annurev-arplant-050312-120205 23638826

[82] Beaulieu J, Jean M, Belzile F. The allotetraploid *Arabidopsis thaliana-Arabidopsis lyrata* subsp. *petraea* as an alternative model system for the study of polyploidy in plants. Mol Genet Genomics. 2009;281(4):421–35. 10.1007/s00438-008-0421-7 19148683

[83] Fujimoto R, Taylor JM, Sasaki T, Kawanabe T, Dennis ES. Genome wide gene expression in artificially synthesized amphidiploids of *Arabidopsis*. Plant Mol Biol. 2011;77(4–5):419–31. 10.1007/s11103-011-9820-y 21882042

[84] Akama S, Shimizu-Inatsugi R, Shimizu KK, Sese J. Genome-wide quantification of homeolog expression ratio revealed nonstochastic gene regulation in synthetic allopolyploid *Arabidopsis*. Nucleic Acids Res. 2014;42(6):e46 10.1093/nar/gkt1376 24423873 PMC3973336

[85] Mariam AL, Zakri AH, Mahani MC, Normah MN. Interspecific hybridization of cultivated rice, *Oryza sativa* L. with the wild rice, O. *minuta* Presl. Theor Appl Genet. 1996;93(5–6):664–71. 10.1007/BF00224060 24162392

