# Supplementary figures and images for: Recreating Stable Brachypodium hybridum Allotetraploids by Uniting the Divergent Genomes of B. distachyon and B. stacei
Source: PLoS One. 2016 Dec 9;11(12):e0167171. doi: 10.1371/journal.pone.0167171 (PMC5147888; doi:10.1371/journal.pone.0167171)

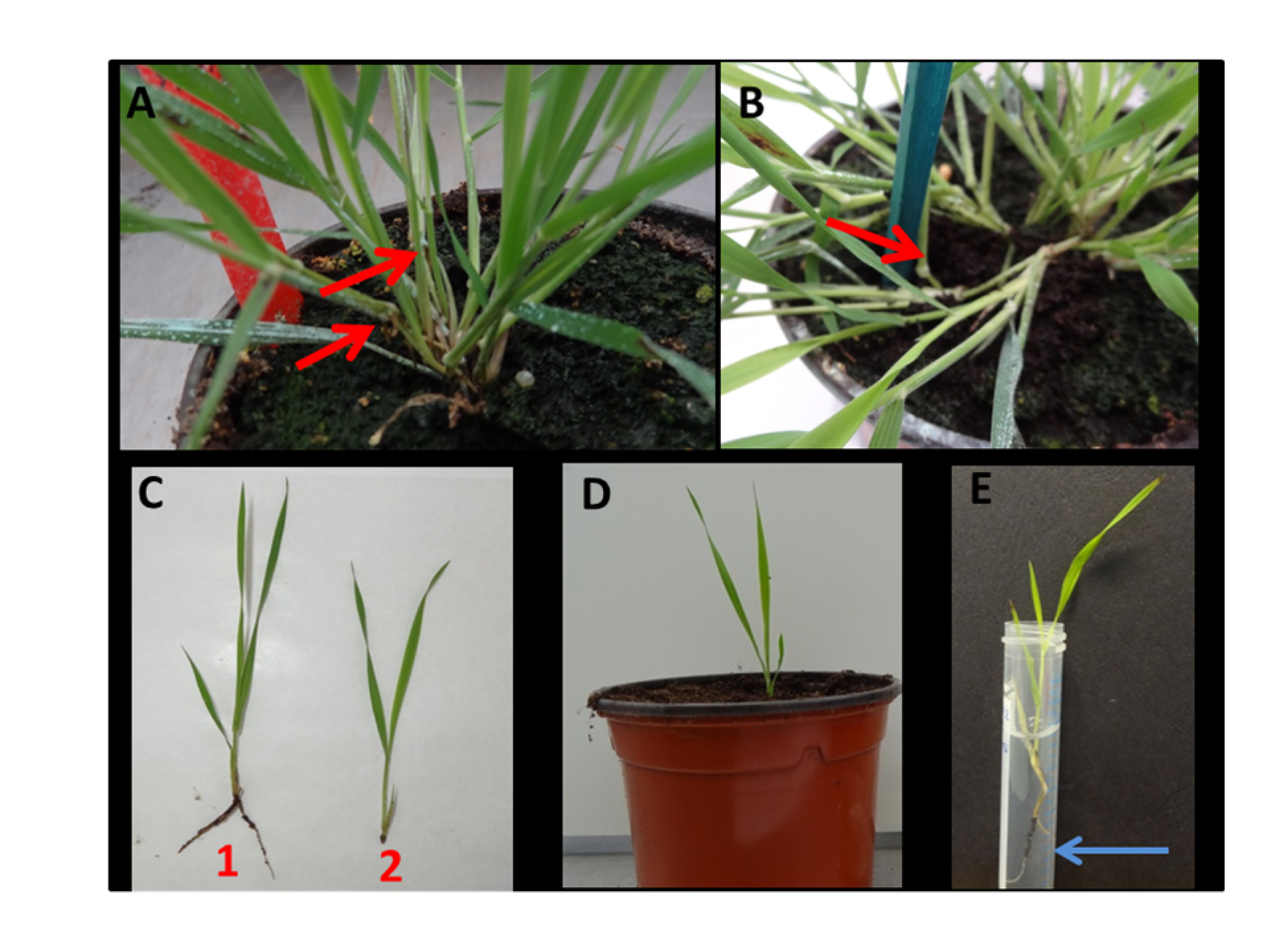

Supplement: S1 Fig — (A) Tillers with secondary roots emerging from the node (indicated by arrows) were used for propagation. (B) Stimulation rooting by burying tiller node in the soil containing 0.25% indole-3-butyric Acid (IBA). The tiller was held in place with a stick. (C) Rooted tillers cut from the initial plant: (1) A tiller with enough roots to live independently, (2) A tiller with no roots. (D) The rooted tiller was transferred directly into a pot to produce a new plant. (E) The rootless tiller was placed in water containing 0.25% IBA. After 7–10 days, the root emerged (indicated by blue arrows) and grew enough to transfer this tiller into a new pot. (TIF) [file pone.0167171.s001.tif]

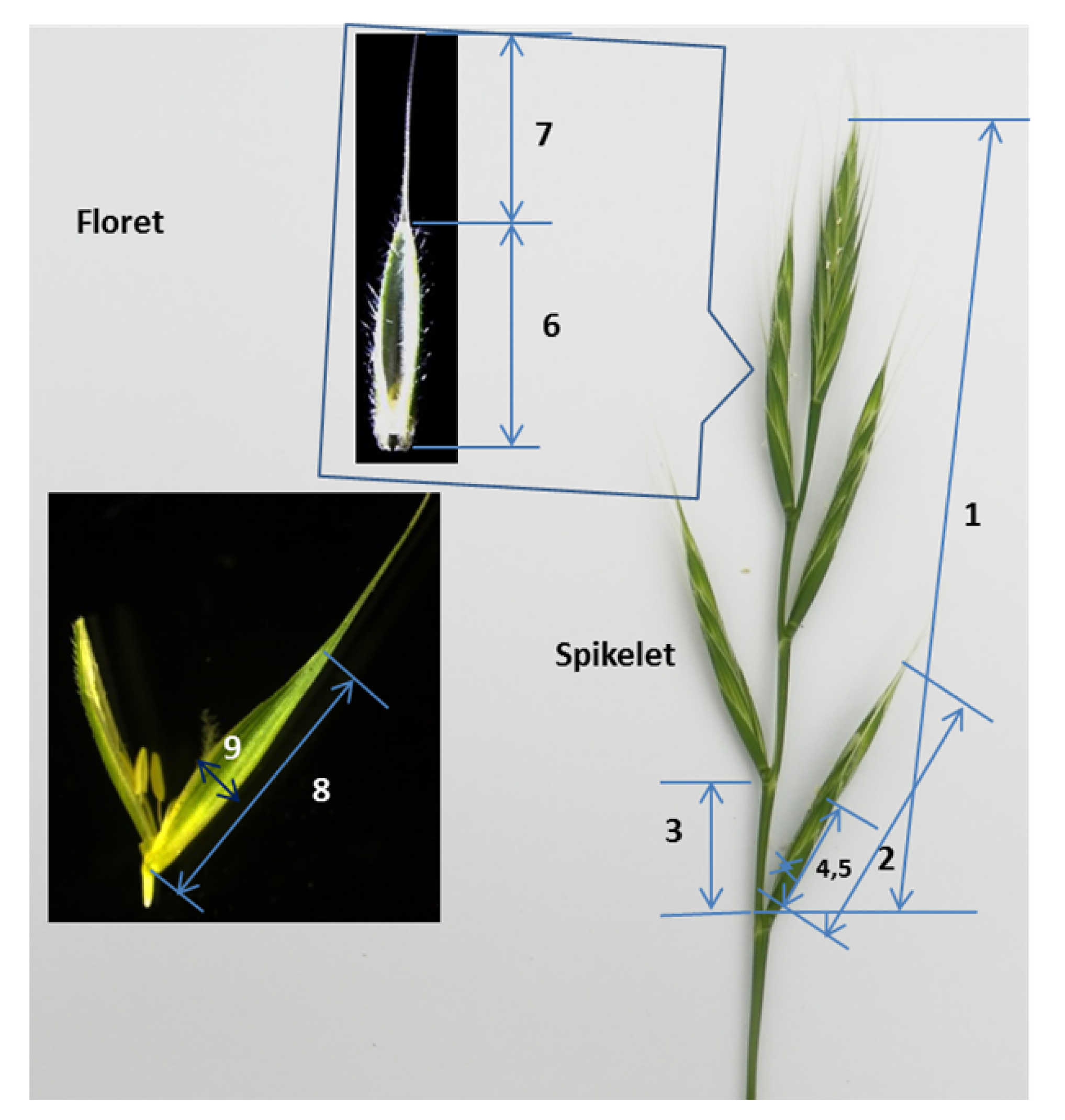

Supplement: S2 Fig — 1. Inflorescence length; 2. Spikelet length; 3. Distance between spikelets; 4. Upper glume from basal spikelet length; 5. Upper glume from basal spikelet width; 6. floret length (the second floret of all spikelets on one inflorescence were measured); 7. Awn length; 8. Lemma length; 9. Lemma width. Other characters measured for inflorescence or spikelet: 10. Spikelet number per inflorescence (all spikelets in the spike—5 in this example); 11. Floret number per spikelet (i.e. number of florets in each spikelet); 12. Floret number per inflorescence (i.e. all florets in an inflorescence); 13. Seed number per inflorescence; 14. Percentage of fertile florets; 15. Weight of 1,000 (TIF) [file pone.0167171.s002.tif]

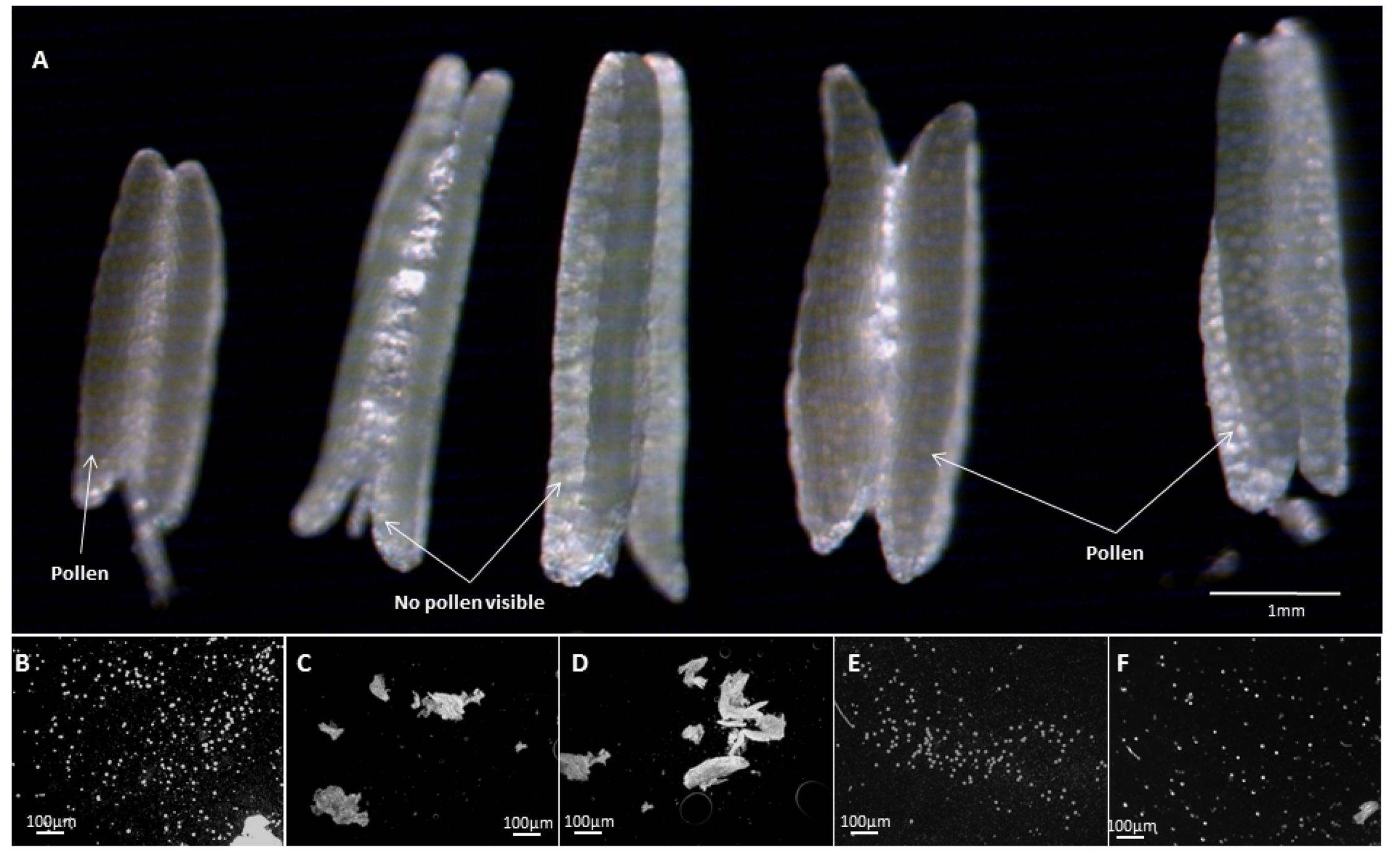

Supplement: S3 Fig — (A) Anthers on the day of anthesis. (B) Spontaneous release of the pollen from anthers after 15–20 minutes incubation on a microscope slide: (B) Bd21, (C) ABR113 and (D) ABR114. This phenomenon was not observed for (E) F1_21×114 and (F) S1 generation of allo21×114; after macerating these anthers there was very little pollen compared with progenitors and natural polyploid. (TIF) [file pone.0167171.s003.tif]
