# Supplementary material for: Recreating Stable Brachypodium hybridum Allotetraploids by Uniting the Divergent Genomes of B. distachyon and B. stacei
Source: PLoS One. 2016 Dec 9;11(12):e0167171. doi: 10.1371/journal.pone.0167171 (PMC5147888; doi:10.1371/journal.pone.0167171)
Supplement: S1 Table — (DOCX) [file pone.0167171.s004.docx]

**S1 Table.** Vegetative multiplication of amphihaploid F1 interspecific hybrids and their treatment with varying concentrations of colchicine (2.5 g/l, 5g/l and 7.5 g/l).

| **F1 interspecific amphihaploid hybrids** | **Number of  multiplied plants** | **Number of flowering plants** | | **Number of plants  treated with colchicine and (surviving ones)** | | |
| --- | --- | --- | --- | --- | --- | --- |
|  |  |  |  | **2.5 g/l** | **5g/l** | **7.5g/l** |
| **F1_21×114.1** | 75 | 24 | | 7 (5) | 32 (28) | 12 (4) |
| **F1_21×114.2** | 124 | | 46 | 15 (12) | 46 (39) | 17 (6) |
| **F1_21×114.3** | 59 | | 11 | 11 (7) | 25 (19) | 12 (2) |
| **F1_21×114.4** | 64 | | 16 | 10 (8) | 25 (22) | 13 (1) |
| **F1_3-1×5** | 3 | | 2 | 0 (0) | 1 (1) | 0 (0) |
| **Total** | **325** | | **99** | **43** | **129** | **54** |
