# Supplementary material for: Recreating Stable Brachypodium hybridum Allotetraploids by Uniting the Divergent Genomes of B. distachyon and B. stacei
Source: PLoS One. 2016 Dec 9;11(12):e0167171. doi: 10.1371/journal.pone.0167171 (PMC5147888; doi:10.1371/journal.pone.0167171)
Supplement: S4 Table — (DOCX) [file pone.0167171.s007.docx]

**S4 Table.** Summary of comparison of some floral characters at the anthesis stage (with dissecting microscope) between *B. distachyon, B. stacei*, *B. hybridum,* interspecific F1 hybrids and S1 generation synthetic allopolyploids allo21×114 and allo3-1×5 plants.

| **Characters** | ***B. distachyon***  (Bd21 and Bd3-1) | ***B. stacei***  (ABR114 and Bsta5) | ***B. hybridum***  (ABR113 and Bhyb30) | **Interspecific hybrid** (F1_21×114  and F1_31×5) | **Synthetic allopolyploid** (allo21×114  and allo3-1×5) |
| --- | --- | --- | --- | --- | --- |
| Anther number | 2 | 3 | 3 | 3 | 3 |
| Anther size^a^ | Small | Bigger | Bigger | Bigger | Bigger |
| Stigma ^a^ | Very feathery | Less feathery | Less feathery | Less feathery | Less feathery |
| Anther dehiscence of the first floret in the spike | 6-7 days from the spike emergence | 4-5 days from the spike emergence | 6-7 days from the spike emergence | 6-7 days from the spike emergence | 6-7 days from the spike emergence |
| Paleas and lemmas | Plump oval shape, much hairy in border of paleas and outside surface of lemmas | Elongated oval shape, very few even do not have hairs in the border of paleas and outside surface of lemmas | Elongated oval shape, few hairy in border of paleas and outside surface of lemmas | Elongated oval shape, few hairy in border of paleas and outside surface of lemmas | Elongated oval shape, few hairy in border of paleas and outside surface of lemmas |

^a^ See Fig. 4E for anther and stigma size and shapes
