# Supplementary material for: Recreating Stable Brachypodium hybridum Allotetraploids by Uniting the Divergent Genomes of B. distachyon and B. stacei
Source: PLoS One. 2016 Dec 9;11(12):e0167171. doi: 10.1371/journal.pone.0167171 (PMC5147888; doi:10.1371/journal.pone.0167171)
Supplement: S5 Table — (DOCX) [file pone.0167171.s008.docx]

**S5 Table.** Number of crosses made between the diploid parental species *B. distachyon* and *B. stacei* as well as *B. hybridum* natural allopolyploids with the interspecific hybrid F1_21×114 and the synthetic allopolyploid allo21×114 (at the S1 generation).

| **♂**  **♀** | ***B. distachyon***  **2xBd21** | ***B. stacei***  **2xABR114** | ***B. hybridum***  **ABR113** | ***B. hybridum***  **Bhyb30** |
| --- | --- | --- | --- | --- |
| **F1_21×114** | 102 | 95 | 125 | 130 |
| **allo21×114** | 105 | 112 | 136 | 127 |
