# Supplementary material for: Recreating Stable Brachypodium hybridum Allotetraploids by Uniting the Divergent Genomes of B. distachyon and B. stacei
Source: PLoS One. 2016 Dec 9;11(12):e0167171. doi: 10.1371/journal.pone.0167171 (PMC5147888; doi:10.1371/journal.pone.0167171)
Supplement: S1 Text — (DOCX) [file pone.0167171.s009.docx]

**S1 Text.**

**Phenotypic characterization of synthetic allopolyploids**

The inflorescence length was larger in *B. stacei* than in both parental lines of *B. distachyon*, whereas they were intermediate to the natural *B. hybridum* allopolyploid lines, a result that is in agreement with the findings of [Catalan, López-Álvarez (1](#_ENREF_1)]. Inflorescence length was similar in both types of synthetic allotetraploids and significantly longer than in *B. distachyon*, averaging values of mid parents (MPVs), and in natural allopolyploids. However, compared to *B. stacei*, the inflorescence length was significantly longer in allo21×114 than in the ABR114 paternal line, whereas it was similar inallo3-1×5 and in the *B. stacei* Bsta5 parental line. The spikelet length, the distance between spikelets, the upper glume width and the floret length exhibit similar comparison trends. All four characters were longer in *B. stacei* than in the *B. distachyon* lines. They were also similar between both synthetic allopolyploids and higher than in *B. distachyon,* MPV, and both lines of the *B. hybridum* natural allopolyploid. Both synthetic allopolyploids were not significantly different from their respective *B. stacei* parent lines for all these traits. Spikelet number per inflorescence, upper glume length and lemma length and width were similar between all compared *Brachypodium* natural species and synthetic allopolyploids. The number of florets per spikelet and number of florets per inflorescence were similar in *B. distachyon* Bd21 and *B. stacei* ABR114 parental lines but significantly lower than in other parental lines, i.e. Bd3-1 and Bsta5. In both synthetic allopolyploids allo21×114 and allo3-1×5 they were significantly larger than in their respective parental lines and in the average value of parents. Awn length was the only trait significantly larger in *B. distachyon* than in *B. stacei*. Both synthetic allopolyploids were more similar to *B. distachyon*, with average values larger than those of mid-parents values and equal to those of natural allopolyploids. The average seed weight was not different between Bd21, ABR114 and natural allopolyploid, but significantly higher in Bsta5 and in allo3-1×5 synthetic allopolyploid than in natural *B. hybridum* and Bd3-1. Regarding the two remaining traits indicative of fertility, seed number per inflorescence (SI) and percent of fertile florets, allo21×114 was sterile and allo3-1×5 was fertile but its fertility rate of 23% was significantly lower than those of the natural *B. hybridum* lines ABR113 (91%) and Bhyb30 (68%).

1. Catalan P, López-Álvarez D, Bellosta C, and Villar L. Updated taxonomic descriptions, iconography, and habitat preferences of Brachypodium distachyon, B. stacei, and B. hybridum (Poaceae). Anales del Jardín Botánico de Madrid. 2016;73(1). doi: http://dx.doi.org/10.3989/ajbm.2428.
