# Supplementary material for: Recreating Stable Brachypodium hybridum Allotetraploids by Uniting the Divergent Genomes of B. distachyon and B. stacei
Source: PLoS One. 2016 Dec 9;11(12):e0167171. doi: 10.1371/journal.pone.0167171 (PMC5147888; doi:10.1371/journal.pone.0167171)
Supplement: S2 Text — (DOCX) [file pone.0167171.s010.docx]

**S2 Text:**

**Classification and interpretation of marker polymorphism**

Monomorphic markers (M) amplify both parental alleles at similar sizes (Fig. 8A) and allowed us to detect genomic rearrangements in the synthetic allopolyploid only if both alleles are deleted or if one of them change in size by deletion or insertions.

By contrast polymorphic markers allow us to characterize the synthetic allopolyploids in different ways. The dominant type or presence/absence polymorphic (PAP) markers, amplify an allele from one parent but not from the other (Fig. 8B) and facilitate the observation of inheritance of only one-parental allele. The codominant type or allele specific polymorphic (ASP) markers, amplify both parental alleles at two different (polymorphic) sizes (Fig. 8C) and allow the characterization of both parental alleles in hybrids and allopolyploids. As recommended in [Mestiri *et al.* (2010](#_ENREF_2)), we separate co-dominant markers that did not amplify appropriately because of technical problems (ASP-), such as competition to PCR amplification (Fig. 8D), using a mixture of parental DNA as a control.
